# Leaf rolling in bread wheat (*Triticum aestivum* L.) is controlled by the upregulation of a pair of closely linked/duplicate zinc finger homeodomain class transcription factors during moisture stress conditions

**DOI:** 10.3389/fpls.2022.1038881

**Published:** 2022-11-22

**Authors:** Ajay Kumar Chandra, Shailendra Kumar Jha, Priyanka Agarwal, Niharika Mallick, M. Niranjana, Vinod

**Affiliations:** Division of Genetics, ICAR-Indian Agricultural Research Institute, New Delhi, India

**Keywords:** leaf rolling, wheat, candidate genes, ZHD TFs, upregulation, moisture stress

## Abstract

Zinc finger-homeodomain (ZF-HDs) class IV transcriptional factors (TFs) is a plant-specific transcription factor and play a key role in stress responses, plant growth, development, and hormonal signaling. In this study, two new leaf rolling TFs genes, namely *TaZHD1* and *TaZHD10*, were identified in wheat using comparative genomic analysis of the target region that carried a major QTL for leaf rolling identified through multi-environment phenotyping and high throughput genotyping of a RIL population. Structural and functional annotation of the candidate *ZHD* genes with its closest rice orthologs reflects the species-specific evolution and, undoubtedly, validates the notions of remote-distance homology concept. Meanwhile, the morphological analysis resulted in contrasting difference for leaf rolling in extreme RILs between parental lines HD2012 and NI5439 at booting and heading stages. Transcriptome-wide expression profiling revealed that *TaZHD10* transcripts showed significantly higher expression levels than *TaZHD1* in all leaf tissues upon drought stress. The relative expression of these genes was further validated by qRT-PCR analysis, which also showed consistent results across the studied genotypes at the booting and anthesis stage. The contrasting modulation of these genes under drought conditions and the available evidenced for its epigenetic behavior that might involve the regulation of metabolic and gene regulatory networks. Prediction of miRNAs resulted in five Tae-miRs that could be associated with RNAi mediated control of *TaZHD1* and *TaZHD10* putatively involved in the metabolic pathway controlling rolled leaf phenotype. Gene interaction network analysis indicated that *TaZHD1* and *TaZHD10* showed pleiotropic effects and might also involve other functions in wheat in addition to leaf rolling. Overall, the results increase our understanding of *TaZHD* genes and provide valuable information as robust candidate genes for future functional genomics research aiming for the breeding of wheat varieties tolerant to leaf rolling.

## Introduction

In recent decades, the improvement of yield and yield-related traits has remained one of the key objectives for the breeding of important crops. The enhancement of photosynthesis is an imperative approach to effusively exploit efficient crop yields (Gao et al., 2019). The leaf blade is the primary organ of photosynthesis and home of dry matter accumulation in crops. Main leaf functions, such as respiration, transpiration, and photosynthesis, rely on three-dimensional architecture of leaf (Zhang et al., 2009). Leaf rolling (LR) can transform the crops’ photosynthetic efficiency and light condition (Gao et al., 2019). For example, low to moderate leaf rolling in *Oryza sativa* (rice) and *Zea mays* (maize) can optimize light transmission and higher canopy photosynthetic efficiency, which can significantly improve drought stress responses by radiant heat absorption and reduction in water loss *via* transpiration, thereby hoarding grain yield (Zhang et al., 2009). In contrast, severe leaf rolling could hinder the leaf’s function and plant development and significantly reduce the yield enactment of the crops.

Zinc finger-homeodomain (ZHD) proteins, a benevolent of plant-specific transcription factors, influence various important biological processes in plants (Khatun et al., 2017; Abdullah et al., 2018; Liu et al., 2021). ZHD proteins contain a C_2_H_2_-type zinc finger (ZF) and homeodomain (HD) domains, which can regulate the expression of target regulatory genes by specific binding to *Cis-*acting regulatory elements (Wang et al., 2016). The first ZHD protein/genes clusters were identified in Flaveria, a C_4_ plant encoding *PEPCase* genes (Windhovel et al., 2001). Subsequently, several plant-specific *ZHD* family genes were identified in various plant species such as *Glycine max* (Deng et al., 2002), *Arabidopsis thaliana* (Hu et al., 2008), *Triticum aestivum* (Bhattacharjee and Jain, 2013), *Oryza sativa* (Xu et al., 2014; Yoon et al., 2020), *Brassica rapa* (Wang et al., 2016), *Solanum lycopersicum* (Khatun et al., 2017), *Gossypium hirsutum* (Abdullah et al., 2018), and *Fagopyrum tataricum* (Liu et al., 2019). Many *ZHD* family genes are involved in vascular development, light signaling, phytohormonal signaling, and biogenesis of plant organs. At the same time, some *ZHD* genes are reported to have a regulatory role in the floral organ development and abiotic stress (salt and dehydration) responses (Wang et al., 2016; Abdullah et al., 2018). For example, the expression level of *AtZHD1* is modulated by abscisic acid, salt, and dehydration stressors (Wang et al., 2016). In addition, NAC proteins can interact with HD proteins that can simultaneously overexpress *NAC* and *ZHD* as a potential regulator of drought tolerance in Arabidopsis (Hu et al., 2008). Further, Hu et al. (2008) also reported that overexpression of *MIF1* interferes with the normal functioning of ZHD proteins. Additionally, *GmZHD1* and *GmZHD2*, the two soybean (*G. max*) ZHD proteins overexpressed calmodulin isoform 4 (*GmCaM4*) encoding gene against the pathogen infestation (Wang et al., 2016). Studies suggesting *ZHD* gene may play significant roles while defining plant growth, development, stress responses, and leaf rolling in plants.

The epidermis-specific expression of *Outer cell layer 1-5* (*OCL1-5*) encodes a protein containing homeodomain-leucine zipper (HD-Zip) TFs modulate rolled leaf, suggesting an increase in the physiological activity of bulliform cells at adaxial surface of leaf blade (Gao et al., 2019). Moreover, the overexpression of *rice outermost cell-specific gene 5* (*OsRoc5*) (Zou et al., 2011) and *OsRoc8* (Fang et al., 2021), a class IV gene homologous to Arabidopsis *GLABRA2* causes adaxially rolled leaves *via* increased size and number of bulliform cells. In contrast, *OsZHD10* and its closest homolog *AtZHD10*, a homeodomain-leucine zipper Arabidopsis class I gene overexpressed in the adaxial region, induce leaf rolling by increased bulliform cells activity in rice (Kim et al., 2007). Similarly, the overexpression of *OsZHD1* and its closest homolog *OsZHD2*, a zinc finger homeodomain class homeobox TFs induced leaf rolling by increased number and size of the bulliform cells at their abaxial surface (Xu et al., 2014; Zhu et al., 2017; Yoon et al., 2020). A similar role was often displayed by *Zea mays* zinc-finger homeodomain protein 10 (*ZmZHD10*) while defining bulliform cell’s shrinkage during heat, salt, and drought tolerance (Abdullah et al., 2018). Recently, 37 zinc-finger homeodomain proteins (TaZHDs) have been identified in wheat. Gene expression analysis of six core groups of *TaZHD* genes suggested that these may be involved in the regulation of critical biological processes, including cold, salt, and drought stress tolerance as a component mediator of leaf rolling trait (Liu et al., 2021), as reported in other crops like maize and rice (Duan et al., 2018; Gao et al., 2019). The information suggested that these genes/transcription factors (TFs) might be putatively involved in adaxial or abaxial leaf rolling by modulation of bulliform cells. However, while the potential regulatory role of *ZHD* genes/TFs has been extensively studied in Arabidopsis, rice, maize, and other model crops, the molecular basis of *ZHD* genes in leaf rolling and its component trait (drought tolerance) in wheat have not yet been identified.

The allohexaploid nature of cultivated wheat is vital in making bread wheat a superior crop to its ancestors because allelic/loci interactions between sub-genomes might contribute to its higher flexibility for gene expression, which may trigger its wide adaptability under drought stress conditions (Guan et al., 2020). IWGSC’s annotation of an exceedingly complex whole genome sequence of wheat has made it possible to identify the candidate genomic loci (genes) on a genome-wide scale. In this study, we report the identification and characterization of *ZHD* genes, a novel candidate leaf rolling gene(s) containing zinc finger homeodomain class homeobox TFs that might be involved in leaf rolling modulation under drought stress conditions. The information generated through the present study could bridge our knowledge gaps concerning ZHD proteins as a critical regulator of bulliform cells’ activity and their possible molecular networks underlying leaf rolling in these important cereal crops.

## Material and methods

### Plant materials, drought treatment, and growth statistics

In this study, four contrasting recombinant inbred lines (RILs) having extreme contrast for leaf rolling index (LRI) viz., high LR (RIL D-13 and RIL D-65) and low LR (RIL D-7 and RIL D-9) along with their parents (NI5439; drought resilient and HD2012; drought susceptible) (Verma et al., 2020), was used for phenotypic analysis, histological study and gene expression profiling. Each RIL representing 25-30 plants was grown in a polyhouse of the Division of Genetics, ICAR-Indian Agriculture Research Institute, New Delhi (28.6377° N, 77.1571° E, and 228.61 m altitude over mean sea level) under controlled conditions during the natural growing season of 2021-22. The experiment was set up in plastic pots filled with a 3:1 ratio of soil and farmyard manure (FYM), with each test RILs replicated thrice in a completely randomized design. During the experimentation period, the plants were allowed to grow under proper conditions (relative humidity: 75-80%; temperature: 25 ± 5 °C; light and dark cycles: 13 + 11 hours; water up to 60-80% soil capacity) (Dong et al., 2019). Moreover, standard agricultural practices were followed to raise a healthy crop instead of any disease management.

Three independent biological replicates were used for each drought treatment and irrigated. A total of 12-15 plants representing each test RILs were subjected to drought treatment as the plants attained booting (stage 10) and heading (stage 10.5) (Lindsey et al., 2017). At each stage, drought treatment was given for ten days without water, while the irrigated (control) pots were allowed to water at regular intervals. For histological and gene expression study, flag leaves of test RILs were harvested as plants exhibiting rolled leaves. The histological study was done with fresh leaf, while for gene expression studies, harvested samples were rapidly frozen in liquid N_2_ and stored at -80°C until use.

### Histological observation of leaves for rolling

Considering the significance of bulliform cells in defining leaf rolling in plants (Yang et al., 2016), free-hand and semi-thin sections of fully expanded flag leaves were used for histological observation in the present study. Harvested leaves were thoroughly washed with running water for free-hand sections and were sectioned about 5 cm in size from their middle portion. Similarly, semi-thin sections of about 50-100 μm thick were done by using a scalpel blade from its middle portion. Furthermore, thin slices were stained with 0.2% Acetocarmine (Sigma) for 2 minutes at 37°C followed by observation and digital photographs were captured with a light microscope (Nikon Y-TV55, Japan) fitted with Nikon Eclipse H600L camera (Nikon, Japan). At least three independent biological replicates were used for each experiment (Yang et al., 2016).

### Identification of candidate gene

In the previous study, we identified a stable QTL putatively associated with leaf rolling Qlr.nhv-5D.2 on the 5D chromosome, flanked with markers AX-94892575 and AX-95124447 at 338665336 and 410953022 intervals, respectively (Verma et al., 2020). Marker intervals were analyzed for the number and type of genes (transcripts), physical locations, protein domains and families, and other gene (protein) specific features with modified parameters using the EnsemblPlants tool, BioMart (https://plants.ensembl.org/biomart/martview/) (Kinsella et al., 2011). The complete nucleotide and coding sequences of identified genes (transcripts) were retrieved from the IWGSC database, readily available at EnsemblPlants (https://plants.ensembl.org/Triticum_aestivum/Info/Index). Homology (BLASTn) search was performed against the recently released wheat genome assembly using BLAST (https://plants.ensembl.org/Triticum_aestivum/Tools/Blast) (Bolser et al., 2016). Furthermore, the complete nucleotide and coding sequences of candidate genes reportedly involved in leaf rolling in model cereals (*O. sativa* and *Z. mays*) were retrieved from Rice Annotation Project Database, RAP-DB (https://rapdb.dna.affrc.go.jp/) and Maize Genetics & Genomics database, MaizeGDB (https://www.maizegdb.org/), respectively (Sakai et al., 2013; Portwood et al., 2019). Finally, putative wheat genes (transcripts) identified within the QTL intervals were used as the query was cross-checked by NCBI blast (https://blast.ncbi.nlm.nih.gov/Blast.cgi) for data reliability based on higher query coverage, lower E-value, and highest % identity (Johnson et al., 2008).

### Chromosomal distribution of candidate gene

Physical mapping exhibiting specified positions of the identified candidate genes corresponding to wheat chromosome was constructed based on the information generated through EnsemblPlants (https://plants.ensembl.org/Triticum_aestivum/Info/Index) and IWGSC-URGI (https://wheat-urgi.versailles.inra.fr/), wheat genome databases (Bolser et al., 2016; Alaux et al., 2018).

### Structural and functional characterization of candidate gene

The DNA sequence of selected wheat genes (transcripts) was subjected to an ORF finder (https://www.ncbi.nlm.nih.gov/orffinder/) for the prediction of coding DNA sequence (CDS) within the gene (Irene et al., 2002). The structural information such as 5´-UTR, exon, intron, and 3´-UTR of candidate genes and their orthologs was extracted using Gene Structure Display Server 2.0 (http://gsds.gao-lab.org/) (Hu et al., 2015). To discover the structural variation in the genes (proteins), conserved domains and motifs were analyzed using the Conserved Domain Architecture Retrieval Tool (https://www.ncbi.nlm.nih.gov/Structure/lexington/lexington.cgi) and MEME suite version 5.0.5 (http://meme-suite.org/tools/meme), respectively (Bailey et al., 2015; Khatun et al., 2017). We utilized MEME suite with slightly modified parameters, i.e., a) motif width: 6-250 amino acids, b) site distribution per sequence: zero or one, and c) maximum no. of motifs: 50 were selected for motif discovery on candidate ZHD proteins. Putative *Cis*-acting regulatory elements in the promoter regions of the identified genes were predicted using the PlantCARE program (http://bioinformatics.psb.ugent.be/webtools/plantcare/html/), a database search engine (Xu F. et al., 2012; Baldoni et al., 2015). Furthermore, the physicochemical characteristics of identified leaf rolling wheat proteins were anticipated by an ExPASy database server, ProtParam (https://web.expasy.org/cgi-bin/protparam/protparam) (Chandra et al., 2020). Sub-cellular and functional localization of leaf rolling wheat proteins was annotated using an integrative web server, BUSCA (http://busca.biocomp.unibo.it/) (Verma et al., 2020).

### Protein structure analysis

Homology modeling is a powerful tool for chimeric proteins’ structural and functional assignment. To cross-check the significant structural similarity between the proteins and their orthologs, the predicted proteins were superimposed based on available homologous proteins in the SWISS-MODEL database server (https://swissmodel.expasy.org/interactive/4QhJDj/templates/) (Gasteiger et al., 2005). Since, in earlier study we had already reported homology modeling for 3D molecular structure and superposition of predicted TaZHD1 protein (Verma et al., 2020), so in the present investigation we only considered second ZHD protein i.e., TaZHD10 for further comparative protein structure analysis using the homology modeling approach (**Supplementary Figure 1A**). The 3D structure homology models at higher complexity levels of the predicted leaf rolling wheat protein TaZHD10 was modeled using SWISS-MODEL (https://swissmodel.expasy.org/interactive) (Gasteiger et al., 2005). Furthermore, to refine and validate the structure of the predicted models, the Ramachandran plot of each protein model was plotted by analyzing psi (Ψ) and phi (Φ) torsion angels using SWISS-MODEL structure assessment (https://swissmodel.expasy.org/assess/), against amino acid residues in the predicted proteins (Waterhouse et al., 2018).

### Sequence alignment and comparative phylogenetic analysis

Protein sequences of predicted leaf rolling wheat proteins and their orthologs were aligned using T-Coffee (https://www.ebi.ac.uk/Tools/msa/tcoffee/) (Di Tommaso et al., 2011). To explore the evolutionary relationship and functional similarity among annotated proteins, phylogenetic tree topology was constructed using PhyML v3.1 (http://www.phylogeny.fr/simple_phylogeny.cgi), a freely available software package (Guindon et al., 2010). To demonstrate the phylogenetic tree, the Maximum-Likelihood method coupled with 1000 iterations of bootstrapping values was used (Wei et al., 2020).

### Prediction of miRNAs targeting candidate genes

Full-length CDS and genomic DNA sequences of identified wheat leaf rolling genes were subjected to a query against an updated version of the wheat miRNAs library, freely available on psRNATarget v2.0 (https://www.zhaolab.org/psRNATarget/analysis?function=2), a plant small RNA target analysis server (Dai et al., 2018). Following default parameters rooted in the psRNATarget algorithm were used likely no. of top targets: 200, expectation: 5, the penalty for G: U pair: 0.5, the penalty for mismatches: 1, seed region: 2-13 nt, extra weight in seed regions: 1.5, no. of mismatch allowed in seed regions: 2, HSP (complementarity scoring) size: 19 and translation inhibition range: 10-11 nt (Liebsch and Palatnik, 2020).

### Differential expression of candidate ZHD gene

#### Transcriptome-wide expression profiling

For estimation of transcript abundance and expression profile of the identified leaf rolling genes, the freely available RNA-seq expression data files (RefSeq v2.1) of wheat (Chinese Spring) were used (Zhu et al., 2021). The relative expression of each gene under drought stress in various tissues at different developmental stages was presented as a heat map. The heat map was generated from the relative abundance of transcripts of each gene, calculated as FPKM (Fragments Per Kilobase of transcript per 10 million reads) using the wheat expression browser (http://www.wheat-expression.com), powered by expVIP (Borrill et al., 2016).

#### RNA isolation, cDNA preparation, and quantitative real-time PCR (qRT-PCR)

The total RNA was extracted from flag leaf tissues of test samples at two different developmental stages, viz., booting (stage 10) and heading (stage 10.5) stage, using the Trizol solution (Invitrogen). Total RNA was treated with RNase-free DNase I (Fermentas) to remove residual DNA contamination. The first-strand cDNA was generated from DNase-treated total RNA (2.5 µg/µl) with oligo (dT) primers using High-capacity cDNA reverse transcription kit (Applied Biosystems). qRT-PCR was performed using GoTaq qPCR master mix (Promega) on a Bio-Rad CFX96 Real-Time system (CA, USA). A set of gene-specific primers were designed using the NCBI Primer-BLAST tool (https://www.ncbi.nlm.nih.gov/tools/primer-blast/) (Untergasser et al., 2012) and commercially synthesized by Eurofins Genomics, India (**Supplementary Table 1**). All test samples were analyzed in three independent biological replications and three technical replicates. The *TaActin1* (LOC123179078), a typical constitutively expressed wheat housekeeping gene was used as an internal control to normalize the expression of candidate genes. The relative expression levels of *ZHD* genes were analysed using irrigated NI5439 as a reference. The relative fold in expression of each gene concerning cycle threshold was calculated using following formulae (Schmittgen and Livak, 2008), represents the cycle numbers required for the detection of fluorescent signal to minimize the background noise.

For the relative expression of candidate *ZHD* gene in any sample,

a) ΔCt_goi_ = Mean (Ct_goi -_ Ct_actin_), here, goi = Gene of interest

b) ΔΔCt = ΔCt_goi (soi)_ - ΔCt_goi (irrigated NI5439) _ soi = Sample of interest

c) Exp (2^ - ΔΔCt) = POWER (2, - ΔΔCt) Ct- value = Cycle threshold

### Gene interaction network analysis

Gene co-expression, co-localization, genetic interaction, as well as physical and shared protein domain analysis, are helpful tools for predicting the biological network integration of predicted genes (Yun et al., 2010; Perrella et al., 2018). In the case of wheat, there is no specific database server for protein-protein, gene-gene, and molecular interaction network study. As a result, biological network and interactome data from *A. thaliana* were used as a reference for real-time gene interaction network analysis of wheat leaf rolling genes on the GeneMANIA web server (https://genemania.org/) (Warde-Farley et al., 2010).

### Epigenetic aspects of leaf rolling

Epigenetic changes script significant impact to plant genomes as transcriptional imprints to adapt and respond against severe stress conditions by reprogramming gene expression (Mohanty et al., 2012; Yong-Villalobos et al., 2015). To elucidate the epigenetic regulation, we analyzed the genome-wide DNA methylation patterns of leaf rolling *ZHD* genes under the differential stress condition (irrigated, drought, and 24 HAW) at the different genic regions. In the case of wheat, there is no specific epigenome platform available for methylome study. As a result, methylome dataset from *O. sativa* were used as a reference for integrated epigenetic regulation and comprehensive analysis of wheat *ZHD* genes using the Plant Methylome database web server (https://epigenome.genetics.uga.edu/PlantMethylome/), powered by JBrowse 1.12.5 (Buels et al., 2016).

## Results

### Histological observation for leaf rolling

**Figure 1:**
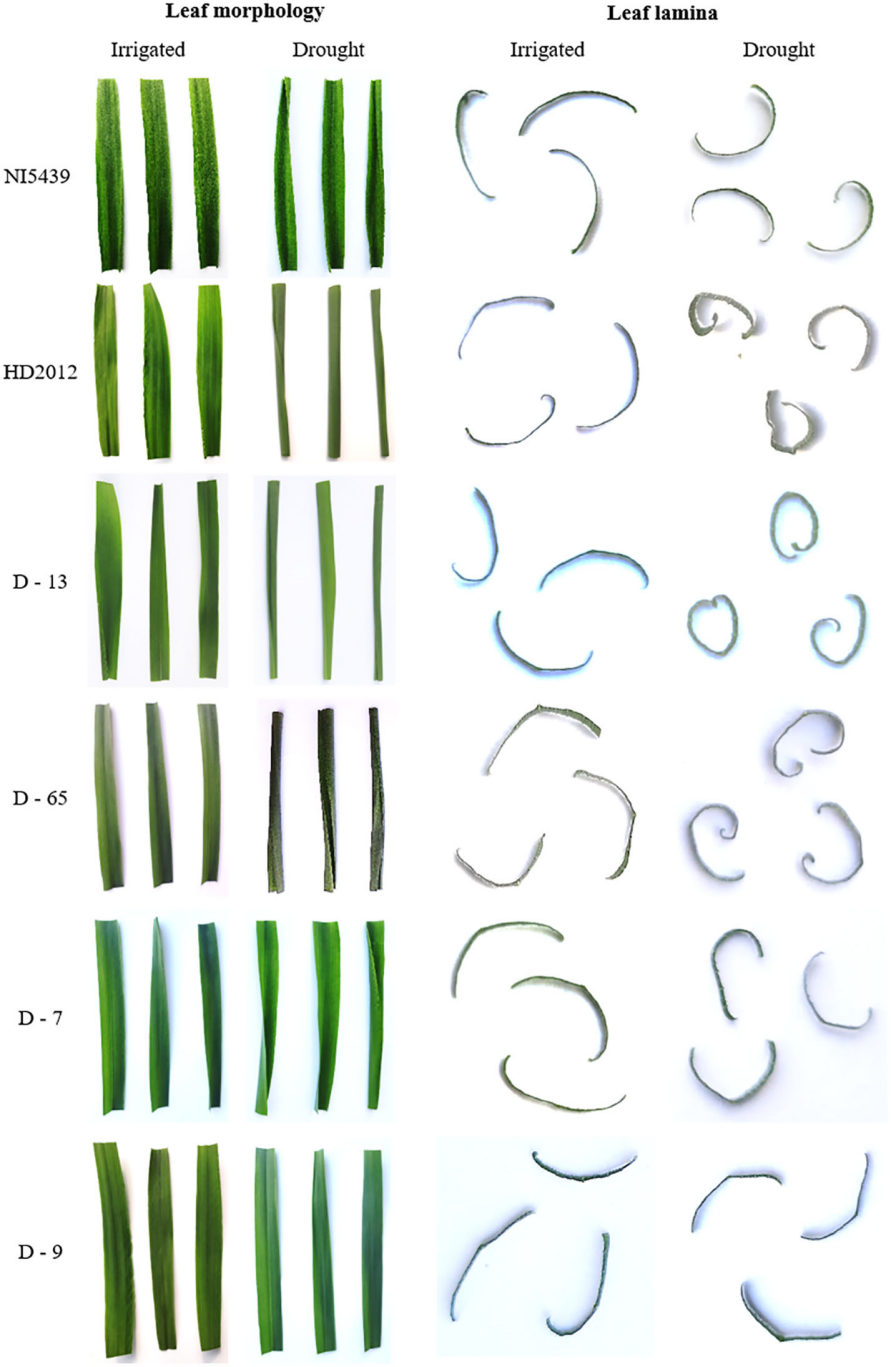
Morphological observations of flag leaf of wheat RILs with parents for rolling (From top to bottom: NI5439; drought resilient, HD2012; drought susceptible, high LR RILs = D-13 and D-65, and low LR RILs = D-7 and D-9).

**Figure 2:**
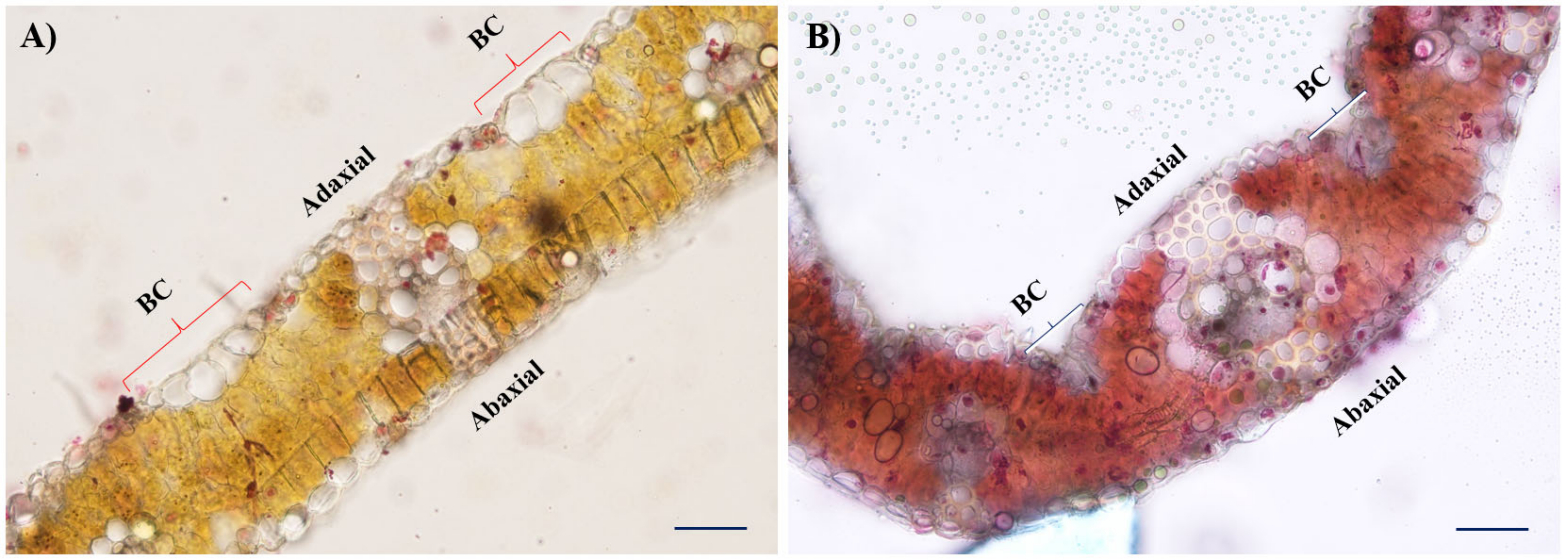
Histological observation of flag leaves contrasting in their propensity for bulliform cells (BC) activity in leaf rolling under moisture stress. **(A)** Cross-sections of RIL D-65 under irrigated condition, and **(B)** Cross-sections of drought treated RIL (D-65) leaves under 20X resolution. Scale bars: 50 µm.

Morphological observation of leaves for rolling resulted in variable architectural differences in drought-treated plants than in the irrigated (control). The gross morphology of wheat genotypes studied under irrigated and drought conditions at the late heading stage is presented in **Figure 1**. At booting (stage 10), all drought-treated plants exhibited semi-roll to completely rolled leaves with significant erectness, owing to reduced leaf lamina joint angle while irrigated plants showed drooping type and flat leaves with no rolling. Similarly, the pattern of the architecture of leaves was almost similar as observed during the booting stage, but the relative intensity of flatness and rolling was significantly higher at the early to late heading (stage 10.5) stage. The relative flatness and rolling of RILs with parents were observed as consistent with our earlier reports. The calculated leaf rolling index (LRI) substantially varied from higher rolling (RIL D-13 and RIL D-65) to low rolling (RIL D-7 and RIL D-9) RILs compared to parents (reported in our earlier studies and documented by Verma et al., 2020). Considering the key role of bulliform cells in defining leaf rolling in wheat, histological analysis was done in present study. We observed that the bulliform cells in irrigated RIL D-65 leaf blades are arranged in a “U” shape and were fully flaccid, while the bulliform cells of treated RIL D-65 are arranged in a “V” shape and were tabid (**Figures 2A, B**).

### Identification of candidate gene

In the previous study, we had identified a stable QTL putatively associated with leaf rolling *Qlr.nhv-5D.2* on the 5D chromosome, flanked with markers *AX-94892575* and *AX-95124447* at 338665336 and 410953022 intervals, respectively (Verma et al., 2020). A total of 5388 transcripts were predicted using a genome-wide analysis approach in this marker interval. Out of 185 unique transcripts, only three viz., *TraesCS5D02G253100.1* and *TraesCS5D02G234600.1*, and *TraesCS5D02G234700.1* speculated to be involved in leaf rolling in *T. aestivum* were identified (**Table 1**). The homology search performed against the already available databases led to the identification of multiple hits. The most significant hits were further shortlisted based on various filters. Genes associated with leaf rolling (*OsZHD1* and *OsZHD10*) from rice showed significant homology with all three transcripts. The transcript *TraesCS5D02G253100.1* showed 96.9% homology and E-value (1.8e-34) with *OsZHD1* (*LOC4347315*) while *TraesCS5D02G234600.1* resulted in 100% homology and E-value (7.8e-149) with *OsZHD10* (*LOC4345673*). In contrast, *TraesCS5D02G234700.1* showed only 40.5% homology and E-value (9.2e-09) with *OsZHD10*, whereas no significant hits were found with *OsZHD1.* Because of this, we excluded our *TraesCS5D02G234700.1* transcript and were not considered for further investigation. Finally, two genes, viz., *TraesCS5D02G253100.1* (*TaZHD1*) and *TraesCS5D02G234600.1* (*TaZHD10*), were considered to be putatively involved in leaf rolling in bread wheat. All the identified genes were annotated according to the name designated to their orthologs. To specify the name of annotated sequences, the first initial of the studied genus (*Triticum*) and species name (*aestivum*) were used in the alias (Ta), followed by the short form (abbreviation) of the protein encoded with their homologs (**Table 1**).

**Table 1:** Identification of candidate genes putatively involved in leaf rolling in wheat and their orthologs.

S. No.	Gene Stable ID*	Gene annotation	Source	Protein type	Genomic Location	Query Length (bp)	E-value	Identity (%)
1.	*LOC4347315* (*LOC_Os09g29130*)	*OsZHD1*	*O. sativa japonica group*	Zinc-finger homeodomain protein 1-like	Chr 9: NC_029264.1 (17703982.17705654)	1673	0.0	87.1
2.	*LOC4345673* (*LOC_Os08g34010*)	*OsZHD10*	*O. sativa japonica group*	Zinc-finger homeodomain protein 10-like	Chr 8: NC_029263.1 (21307444:21309247)	1741	2.00e-80	89.0
3.	*LOC103653493*	*ZmZHD10*	*Z. mays*	Zinc-finger homeodomain protein 10	Chr 4: NC_050099.1 (88731765.88733255)	1491	9.00e-64	84.6
4.	*LOC119309747*	*TdZFH9a*	*T. dicoccoides*	Zinc-finger homeodomain protein 9-like	Chr 5B: NC_041389.1 (418045587.418047238)	1652	0.0	95.2
5.	*LOC119301727*	*TdZFH9b*	*T. dicoccoides*	Zinc-finger homeodomain protein 9-like	Chr 5A: NC_041388.1 (389777977.389779664)	1688	0.0	95.5
6.	*TraesCS5D02G253100.1*	*TaZHD1*	*T. aestivum*	Uncharacterized proteins	5D:359474816-359475706	891	1.8e-34	96.9
7.	*TraesCS5D02G234600.1*	*TaZHD10*	*T. aestivum*	Uncharacterized proteins	5D:342174542-342176173	1632	7.8e-149	100.0

*Gene stable ID obtained from the NCBI or ensemble plants (wheat) database.

### Chromosomal distribution of candidate *ZHD* genes

**Figure 3:**
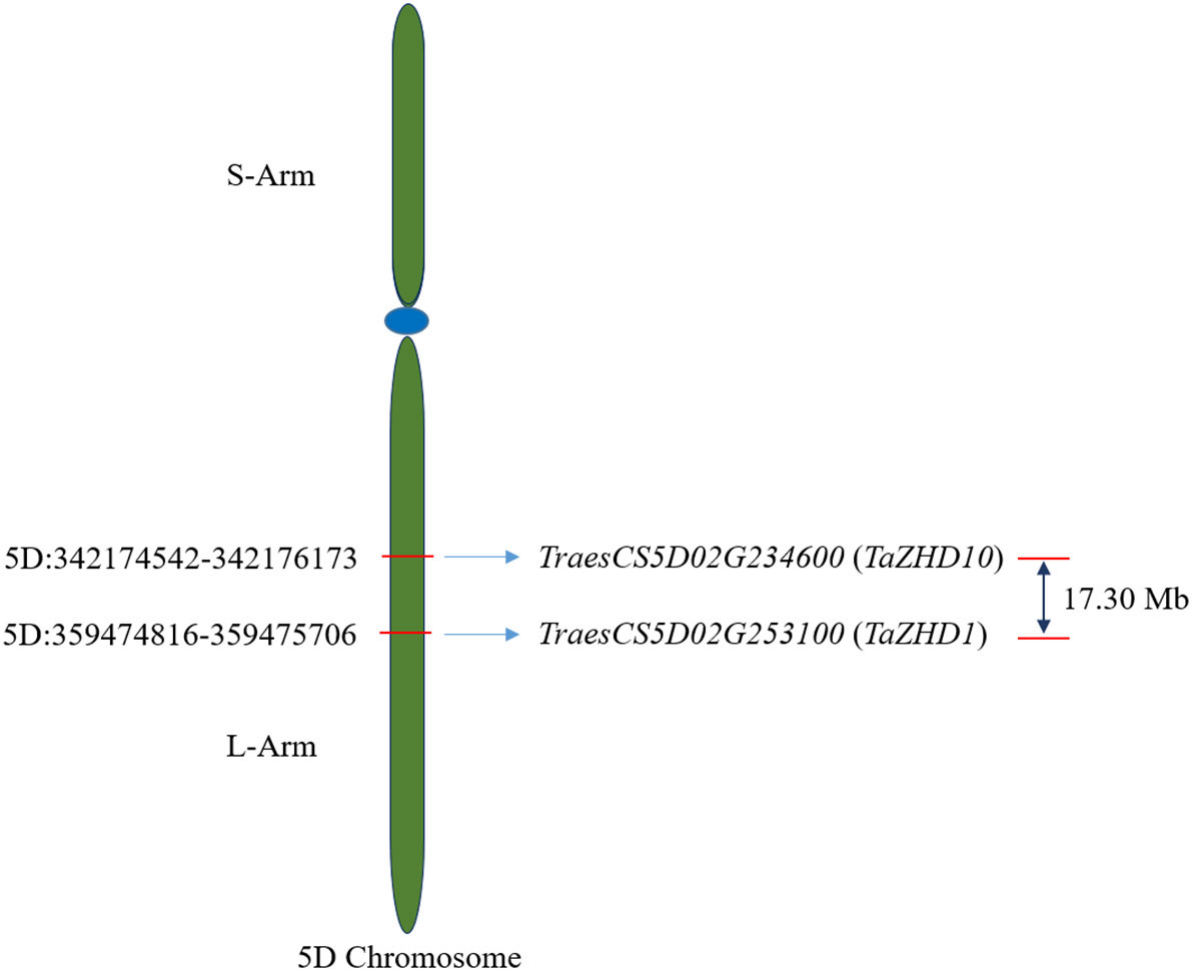
Chromosomal distribution of identified candidate *ZHD* genes in wheat.

The physical positions of identified candidate genes, *TaZHD1* and *TaZHD10*, to corresponding 5D chromosome is depicted in **Figure 3**. A comprehensive study of each gene using the BioMart database server revealed that *TraesCS5D02G253100.1* (*TaZHD1*) is localized on 5D:359474816-359475706 whereas *TraesCS5D02G234600.1* (*TaZHD10*) is confined on 5D:342174542-342176173, at the long arm of the corresponding chromosome. Further, the genetic distance between both genes was calculated using IWGSC-URGI wheat genome databases and was found up to 17.30 Mb, which was considered merely closer to each other.

### Structural and functional annotation of identified ZHD genes

The Open Reading Frame (ORF) or coding DNA sequence (CDS) of each candidate *ZHD* gene was explored by subjecting genomic sequences to an ORF finder. The total number of anticipated ORFs per gene ranged from 5 to 10 ORFs, with an average of eight (8) ORFs. Comprehensive analysis of *TaZHD1* resulted in 9 ORFs, while *TaZHD10* resulted in 5 ORFs with variable lengths. Finally, nucleotide and translated protein sequences of the longest ORFs of each predicted gene were retrieved and used for further structural and functional analysis. The detailed information on best-predicted ORFs, including start, stop, and length statistics, are presented in **Supplementary Table 2**.

**Figure 4:**
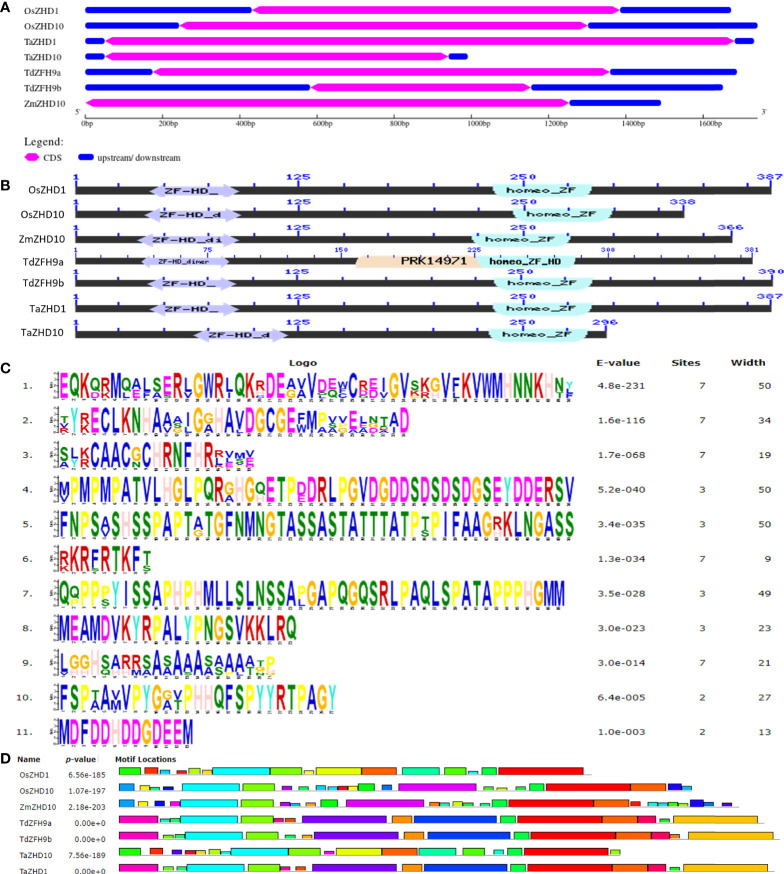
Comparative analysis of **(A)** structural organization of *ZHD* genes, **(B)** conserved domains, **(C)** conserved motifs, and **(D)** locations of motifs, of candidate leaf rolling ZHD protein of orthologs and their best possible match with wheat.

The structural organization of genes, including 5’-UTR, exon-introns pattern, and 3’- UTR contain essential information to understand gene evolution and was predicted by subjecting genomic sequences and CDS to Gene Structure Display Server (**Figure 4A**). The results showed that both *TaZHD1* and *TaZHD10* genes contain a single exon flanked by upstream and downstream sequences, as displayed in their respective homologs. The length of upstream and downstream sequences was almost equal, but exons’ size was highly variable in both genes. The number and lengths of exons were relatively more conserved, suggesting that both genes govern potentially conserved biological functions.

During the comprehensive analysis of physicochemical properties of translated ZHD proteins, the chimeric proteins resulted in a wide array of variability in terms of molecular weight, a total number of amino acids, grand average of hydropathicity (GRAVY), aliphatic index, instability index, and isoelectric point (pI). The characteristic properties revealed that the identified chimeric proteins are physiochemically identical to their respective homologs (**Supplementary Table 2**).

Proteins belonging to the ZF-HD IV family are potential plant-specific transcription factors such as *TaZHD1* and *TaZHD10*. To determine whether *TaZHD1* and *TaZHD10* are found in the nucleus, the subcellular location of these proteins was investigated. *TaZHD1* and *TaZHD10* were found solely in the nucleus, indicating that they are nuclear proteins, according to the results (**Supplementary Table 2**).

### Wheat *ZHD* genes possess zinc finger homeodomain (ZF-HD) IV family transcription factors

Wheat genome annotation analysis using the *EnsemblPlants* database showed that *TaZHD1* and *TaZHD10* are located on the 5D chromosome and encode a 387 and 316 amino acid residue protein, respectively. Multilevel sequence analysis of candidate proteins revealed the presence of two functional domains, ZF-HD dimer superfamily and homeo_ZF_HD superfamily domain designated by cl04737 and cl22771 accession numbers, respectively (**Figure 4B**). Both superfamily domains belong to plant-specific transcription factors and are found to be ubiquitously present in *A. thaliana*, C_4_, and other C_3_ plants while regulating the expression of key genes. Further, based on the similarity index to proteins of known structure, these domains are believed to be involved in forming hetero- and homodimers. They may form zinc fingers, an essential constituent of DNA binding domains.

**Figure 5:**
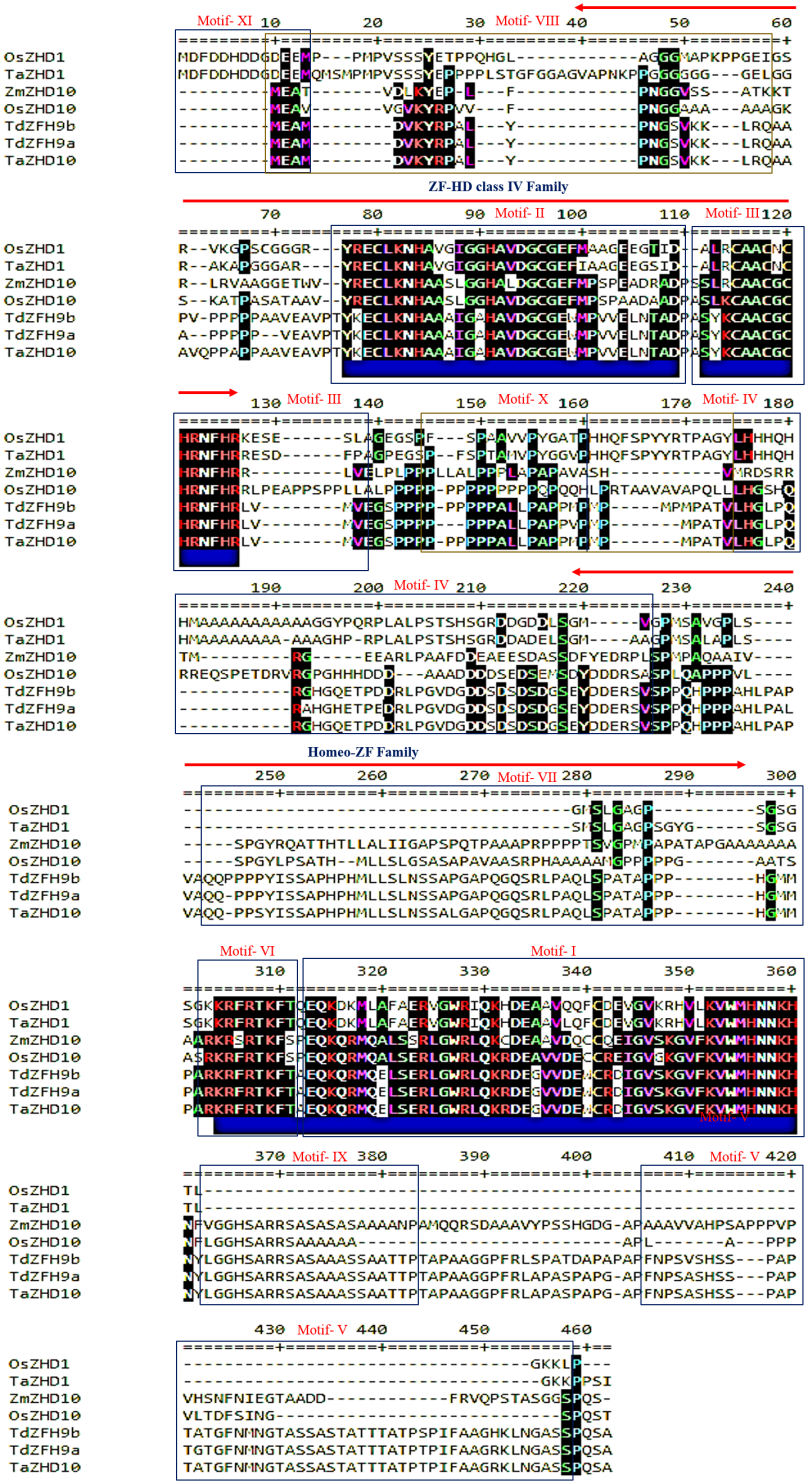
Multiple sequence alignment of the conserved domain and motifs distributed across the candidate leaf rolling *ZHD* genes in wheat and its orthologs.

In addition to this, motif analysis identified a set of 11 conserved motifs distributed across the putative chimeric proteins. The relative distribution of conserved domains and motifs across the putative chimeric proteins are depicted in **Figures 4B–D**, and presented in **Supplementary Table 3**. Out of these, the motif-I, IV, and V are considered to be the longest (50 aa), followed by motif-VII (49 aa) and motif-II (34 aa), whereas motif-VI (09 aa) is designated as the shortest one based on their length of consensus sequences. Further, to localize the specific positions of identified domains and motifs, we aligned protein sequences of identified candidate proteins with their homologs. The results revealed that the ZF-HD dimer superfamily is estimated to be embedded between 40-125 peptides, while the homeo_ZF_HD superfamily domain lies between 220 to 300 peptides as designated in their respective homologs (**Figure 5**). The ZF-HD dimer superfamily was found to share the consensus sequences of motif-VIII (40%), motif-II (100%), and motif-III (80%) while embedded with some undefined regions. Similarly, the homeo_ZF_HD superfamily domain was formed by sharing consensus sequences of motif-IV (10%) and motif-VII (100%) and some undefined regions. Besides this, multilevel sequences analysis revealed that the motif-XI and VIII share some overlapped regions of about four amino acid long consensus sequences while motif-X and IV share 14 amino acid long overlapped regions.

**Figure 6:**
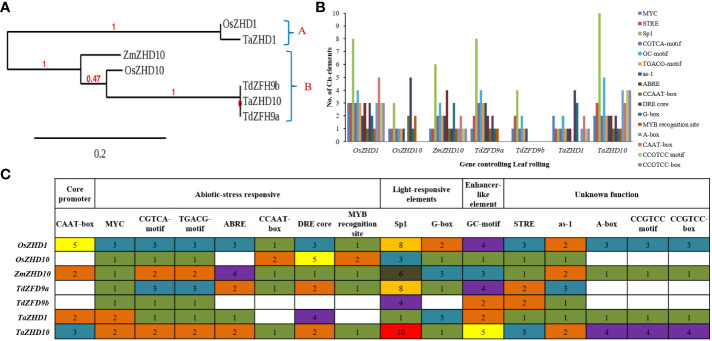
Comparative analysis of, **(A)** phylogenetic tree topology, **(B)** *Cis*- acting regulatory elements in promoters, and **(C)** functional classes of annotated *Cis-*acting regulatory elements of candidate leaf rolling *ZHD* genes of wheat and their orthologs (*Cis*-acting regulatory elements identified in genes were represented in the form of bars).

Finally, to explore the evolutionary significance of identified genes (proteins), a phylogenetic tree was constructed using the Maximum-Likelihood method. Based on the higher bootstrap values, the rooted phylogenetic tree topology grouped annotated genes into two phylogenetic clusters viz., clusters A and B (**Figure 6A**). Cluster A includes genes like *OsZHD1* and *TaZHD1*, while cluster B includes genes like *ZmZHD10, OsZHD10, TaZHD10, TdZFH9a*, and *TdZFH9b*. The study showed that *TaZHD1* shares high similarities with other homologs in rice (*OsZHD1*). Likewise, the *TaZHD10* is more closely related to homologs in rice (*OsZHD10*) and emmer wheat (*TdZFH9a* and *TdZFH9b*) but distantly with homologs of maize (*ZmZHD10*). Furthermore, multiple sequence alignment of amino acids showed that *TaZHD1* shares 96.9% identity with *OsZHD1*. Similarly, *TaZHD10* shares a significantly higher identity with rice *OsZHD10* (89.0%) and emmer wheat homologs, including, *TdZFH9a* (95.2%) and *TdZFH9b* (95.5%), while least with maize (84.6%) homologs. Altogether, this information suggested that *TaZHD1* and *TaZHD10* share high homology, a common origin from similar orthologs, and possess the typical features of a class zinc finger homeodomain (ZF-HD) IV family transcription factor/gene.

### Structural analysis of candidate ZHD proteins

Modeled 3D structure of TaZHD10 shared up to 100% similarity with respective homolog. Using an automated Swiss-Model server, the modeled 3D structure was further examined for superposition with acceptable template structures. The TaZHD10 protein was found superposed (<1 Å RMSD) with more than seven ZF-HD homeobox family proteins of *A. thaliana* sharing up to 55.74-60.34% similarity. Among them, the homeobox domain of *A. thaliana* hypothetical protein (F22K18.140) displayed up to 59.65% similarity upon superposition (**Supplementary Table 4**). The similarity of proteins up to this level was believed to be adequate for annotation of the 3D molecular structure of predicted proteins. As per homology modeling and superposition rule offered by automated Swiss-Model server, modeled protein exhibiting more than 30% similarity with template structure is considered an excellent model (**Supplementary Figure 1A**).

To evaluate the quality of modeled 3D structure of TaZHD1 and TaZHD10 protein, we further analyzed the Ramachandran plot of each protein in contrast with template structure (homologs). As depicted in **Supplementary Figure 1B**, phi (Φ) and psi (Ψ) torsion angles of Ramachandran plots revealed an excellent geometry of modeled 3D structure of TaZHD1 and TaZHD10 proteins. Ramachandran plot calculation showed up to 2.53% residues in generous outlier regions and 12.86% residues in rotamer outlier regions. In comparison, up to 86.56% of residues were present in the most favored regions, commonly called Ramachandran regions. The results suggested the excellent quality of modeled proteins as displayed through PROCHECK algorithms, a widely used method to estimate the stereochemical quality of modeled proteins. Comprehensive descriptions of Ramachandran plot structure assessment of identified leaf rolling gene (protein) in wheat with homologs are presented in **Supplementary Table 5**.

### Analysis of *Cis*-acting regulatory elements in *ZHD* genes

In the promoters’ region, the frequency of *Cis-*acting regulatory elements plays a vital role in defining the stress-responsive or tissue-specific expression of a key gene under variable environmental conditions (Khatun et al., 2017). A web-based search was carried out from available databases to identify possible hormones and stress-responsive *Cis-*acting regulatory elements in the promoter regions of wheat *ZHD* genes. The PlantCARE database predicted a set of 34 *Cis*-acting regulatory elements scattered across promoter regions of the putative candidate genes. The result revealed that both candidate genes possess CAAT-box (CAAT) as basal or core promoter and enhancer regions. In contrast, other 33 *Cis*-acting regulatory elements were also found, directly or indirectly involved in biotic & stress-responsiveness, phytohormonal signaling, light-responsiveness, and other developmental processes. During comprehensive analysis, the study revealed that both candidate genes possess large numbers of *Cis*- acting regulatory elements in promoter regions as recorded in their homologs. Because of a large set of identified *Cis*- acting regulatory elements, the study was finally focused on the 16 most frequent *Cis-*acting regulatory elements in promoters. Out of 16, we found seven abiotic-stress responsive *Cis*-acting regulatory elements, including MYC (CATGTG/CAATTG), CGTCA-motif (CGTCA), TGACG-motif (TGACG), ABRE (ACGTG/GCCGCGTGGC), CCAAT-box (CAACGG), DRE core (GCCGAC) and MYB recognition site (CCGTTG), involved in the regulation of drought stress-inducible genes. In addition, CGTCA-motif and TGACG-motif are engaged in the MeJA-responsive expression of genes. *Cis-*acting regulatory elements like Sp1 (GGGCGG) and G-box (TACGTG/CACGAC) play a specific role as light-responsive elements, whereas GC-motif (AGCGCGCCG/CCCCCG) is an enhancer-like element involved in anoxic specific inducibility of target genes. Furthermore, the particular role of other *Cis-*acting regulatory elements like STRE (AGGGG), as-1 (TGACG), A-box (CCGTCC), CCGTCC motif (CCGTCC), and CCGTCC-box (CCGTCC) are yet to be defined.

The *TaZHD1* possessed all 14 *Cis-*acting regulatory elements except CCAAT-box and MYB recognition site, while the *TaZHD10* had all 16 types *Cis-*acting regulatory elements in their promoter regions (**Figure 6B**). Besides, *Cis-*acting regulatory elements like DRE core (4) and G-box (3) exist in many copies in *TaZHD1*, correspondingly the STRE (3), Sp1 (10), GC-motif (5), A-box (4), CAAT-box (3), CCGTCC motif (4) and CCGTCC-box (4) are present in many copies in *TaZHD10* (**Figure 6C**). Consequently, because of a huge set of stress-responsive *Cis*-acting regulatory elements in promoter regions of *TaZHD1* and *TaZHD10*, the present analysis can conclude that *TaZHD1* and *TaZHD10* are a type of abiotic (drought) stress-inducible genes that might involve in the expression of rolled leaf phenotype under a drought-induced environment in wheat.

### Prediction of miRNAs targeting candidate *ZHD* genes

Earlier studies revealed that the miRNAs could play defining role in leaf differentiation by negative regulation of target gene expression at the post-transcriptional level (Moon and Hake, 2011; Xu et al., 2018). So, to unveil the possible role of miRNAs in leaf rolling identified wheat, *TaZHD1*, and *TaZHD10* were searched against the psRNATarget v2.0 server. The study resulted in 5 wheat miRNAs (Tae-miRs) targeting both candidate genes. Identified 5 wheat Tae-miRs belong to three different miR families (**Supplementary Table 6**). Three miRNAs viz., tae-miR1130b-3p, tae-miR531, and tae-miR9666a-3p were targeting *TaZHD1* representing two different miR families. Correspondingly, two miRNAs viz., tae-miR9664-3p and tae-miR9672b were found to be targeting the *TaZHD10*, also representing two different miR families. The expected (E) value of all miRNAs ranged from 4.5 to 5.0 suggesting greater functional specificity of identified Tae-miRs. Furthermore, all miRNAs also exhibited significant pairwise alignment with their complementary query sequence advocating that the identified genes belong to protein-encoding Tae-miRs, which might be involved in generating functional proteins. Besides this, all predicted Tae-miRs showed cleavage inhibition properties with a multiplicity ratio (1). Thus, the results further demonstrated that identified Tae-miRs might associate with RNA-mediated silencing of *TaZHD1* and *TaZHD10* putatively involved in the metabolic pathway controlling rolled leaf phenotype.

### Differential expression profiling of Wheat *ZHD* genes

#### Transcriptome-wide expression profiling

**Figure 7:**
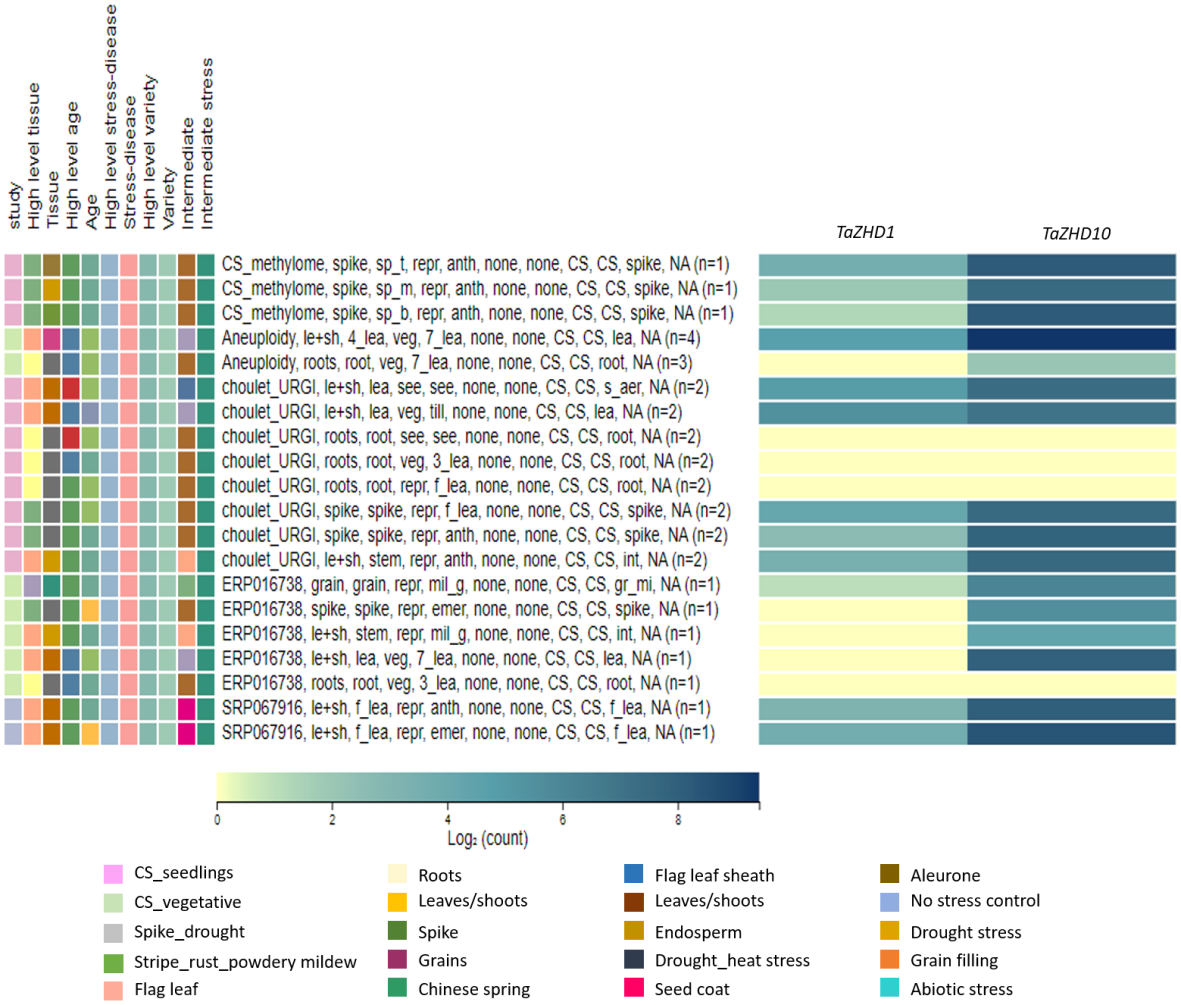
Transcriptome-wide expressions profiling of candidate leaf rolling *ZHD* genes in wheat tissues at different developmental stages and under studied biotic and abiotic stress conditions.

To gain further molecular insights into the relative expression patterns of *TaZHD1* and *TaZHD10* transcripts, we used available RNA-Seq transcriptome datasets for leaf differentiation, plant growth, tissues and organ development, and biotic and abiotic stress responses. Expression profiling of *TaZHD1* and *TaZHD10* transcript resulted in differential expression patterns at three different developmental viz., seedling, vegetative, and reproductive stages. Both transcripts were displayed lower to a higher level of transcript expression in all studied tissues (roots, leaves, shoots, grains, and developmental spikes). Furthermore, *TaZHD1* and *TaZHD10* transcripts showed significantly higher expression levels ranging from 0-10 tpm, in the tissues at seedling, three-leaf stage, tillering, fifth leaf stage, seven leaf stage, booting, flag leaf stage, early-to-late heading, grain filling, and ripening stage. As demonstrated in a heat map (**Figure 7**), both transcripts also showed dramatically higher expression upon biotic (stripe rust, powdery mildew, and fusarium wilt) and abiotic (drought, heat, cold, and phosphorus starvation) stress-induced conditioning wheat, especially in Chinese spring. *In-silico* expression profiling of *TaZHD1* and *TaZHD10* transcripts was further predicted under drought stress compared to control. Expression of *TaZHD1* was significantly reduced under drought stress in tissues of root, stem, 3-7 leaf stage, and developmental spikes compared to irrigated. In contrast, *TaZHD1* was expressed significantly higher in flag leaf and leaf sheath tissues under drought stress at the seedling and reproductive stages. Besides, this *TaZHD10* transcript was predicted to be 2-5 fold up-regulations under drought stress with reference to *TaZHD1.* Similarly, *TaZHD10* was expressed significantly higher in flag leaf, leaf sheath, and developmental spike tissues while reduced in roots at seedling and reproductive stages. Both transcripts showed low to no expression in the roots and 3-leaf stage, while expressed almost equally in flag leaf, leaf-sheath, and spikes throughout all developmental stages (**Figure 7**). Altogether, results indicated that *TaZHD10* showed many fold up-regulation and was ubiquitously expressed in drought-induced leaf tissues, which might also be considered a major component trait of leaf rolling. Henceforth, out of these two transcripts, *TaZHD10* might be the best candidate gene controlling rolled leaf trait under a drought-induced environment in this important cereal crop.

#### Quantitative real-time PCR profiling

**Figure 8:**
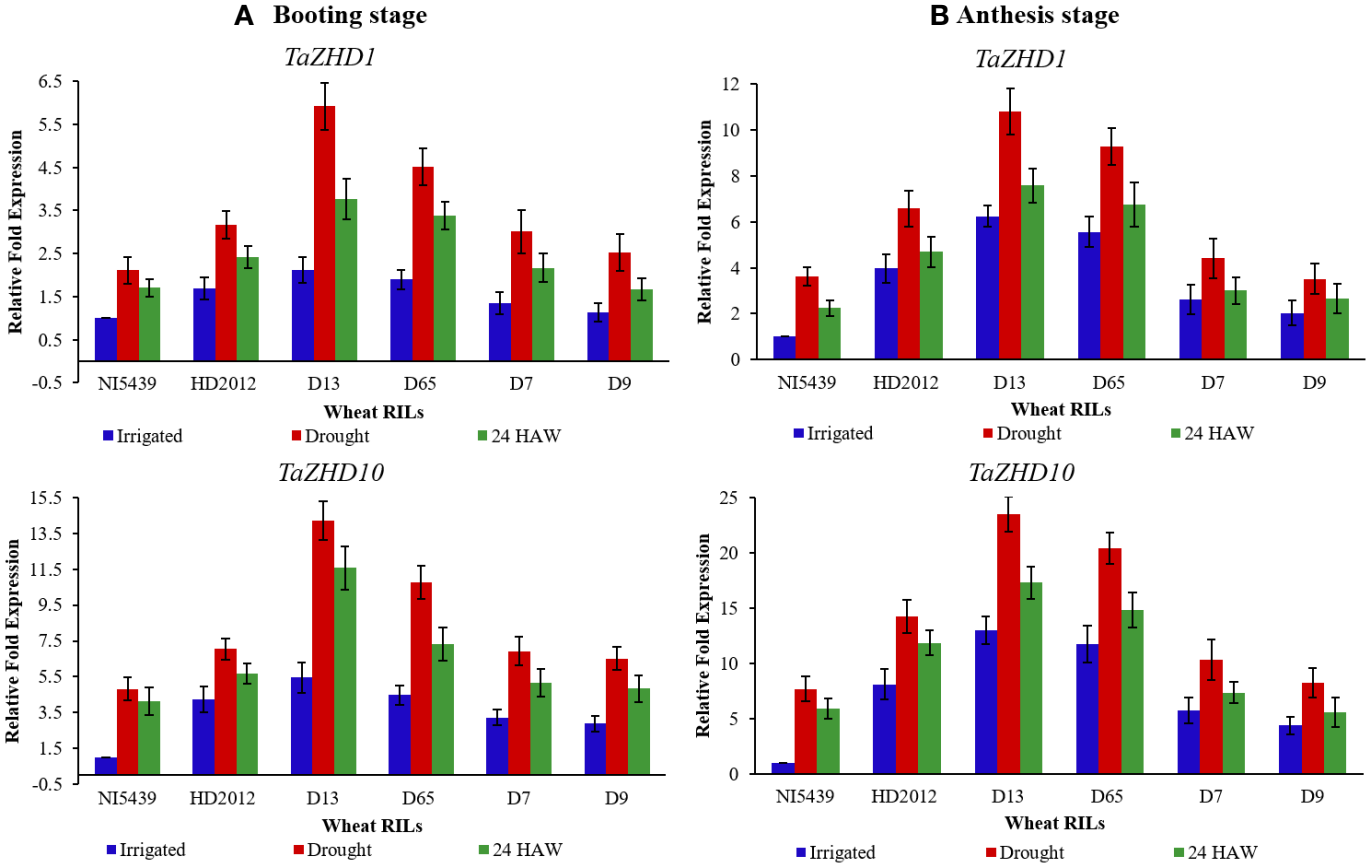
Relative expression profiling of candidate leaf rolling *ZHD* genes in tissues of wheat RILs at **(A)** booting stage, and **(B)** anthesis (heading) stage (From left to right: NI5439; drought resilient, HD2012; drought susceptible, high LR RILs = D-13 and D-65, and low LR RILs = D-7 and D-9; HAW, hours after watering). Each test sample represents three independent biological and three technical replicates.

To further elucidate the functional aspects of identified candidate *ZHD* transcripts we conducted qRT-PCR analysis at two different developmental stages. Likewise, differential expression patterns of these transcripts were also recorded at three different time scales, viz., irrigated, drought (10 days after water withdrawal), and after 24 hours of watering (24 HAW). Both transcripts were variably expressed in flag leaf tissues of all genotypes at both booting (stage 10), and heading (stage 10.5) stages. Under drought conditions, transcripts were initially up-regulated and reached the highest expression level (2-4 folds’ vs irrigated) and then down-regulated (1-3 folds vs drought) upon 24 HAW. Consistent with this, we observed a notable increase/decrease in expression of the *TaZHD1* at a particular growth stage often caused by the up-or down-regulation of *TaZHD10* (**Figures 8A, B**). For example, over-expression of *TaZHD10* was accompanied by the decreased expression of *TaZHD1* at the booting stage and down-regulation of *TaZHD1* caused an increased expression of *TaZHD10* at the heading stage. In contrast, upon drought treatment, the transcript abundance of *TaZHD10* had showed 2-3 folds up-regulation in expression than *TaZHD1* in all studied genotypes.

Overall, the expression pattern we observed for these candidate genes by qRT-PCR analysis was similar to their expression in the obtained RNA-Seq transcriptome data. In addition, differential expression profiles of genes in studied genotypes are highly congruent with the results as observed in histological observations (**Figures 1** and **2**) and leaf rolling index (for more details please follow Verma et al., 2020). Finally, based on the differential expression patterns we hypothesized that there might exist a complex regulatory loop between *TaZHD1* and *TaZHD10* which might control rolled-leaf phenotype. Furthermore, our investigations suggested that the zinc finger homeodomain (ZF-HD) IV family rich proteins might also play an important and complementary role in the transcriptional activator of *TaZHD1* and *TaZHD10* -controlling bulliform cell differentiation. More intriguingly, the study further advocated that the relative expression of candidate transcripts in wheat is highly genotype and treatment-specific, while also varied upon developmental stages. Relative expression profiles of transcripts in tissues of different wheat genotypes studied under irrigated, drought and 24 HAW are presented in **Figures 8A, B**.

### Gene interaction network analysis of *ZHD* genes and other regulatory partners

**Figure 9:**
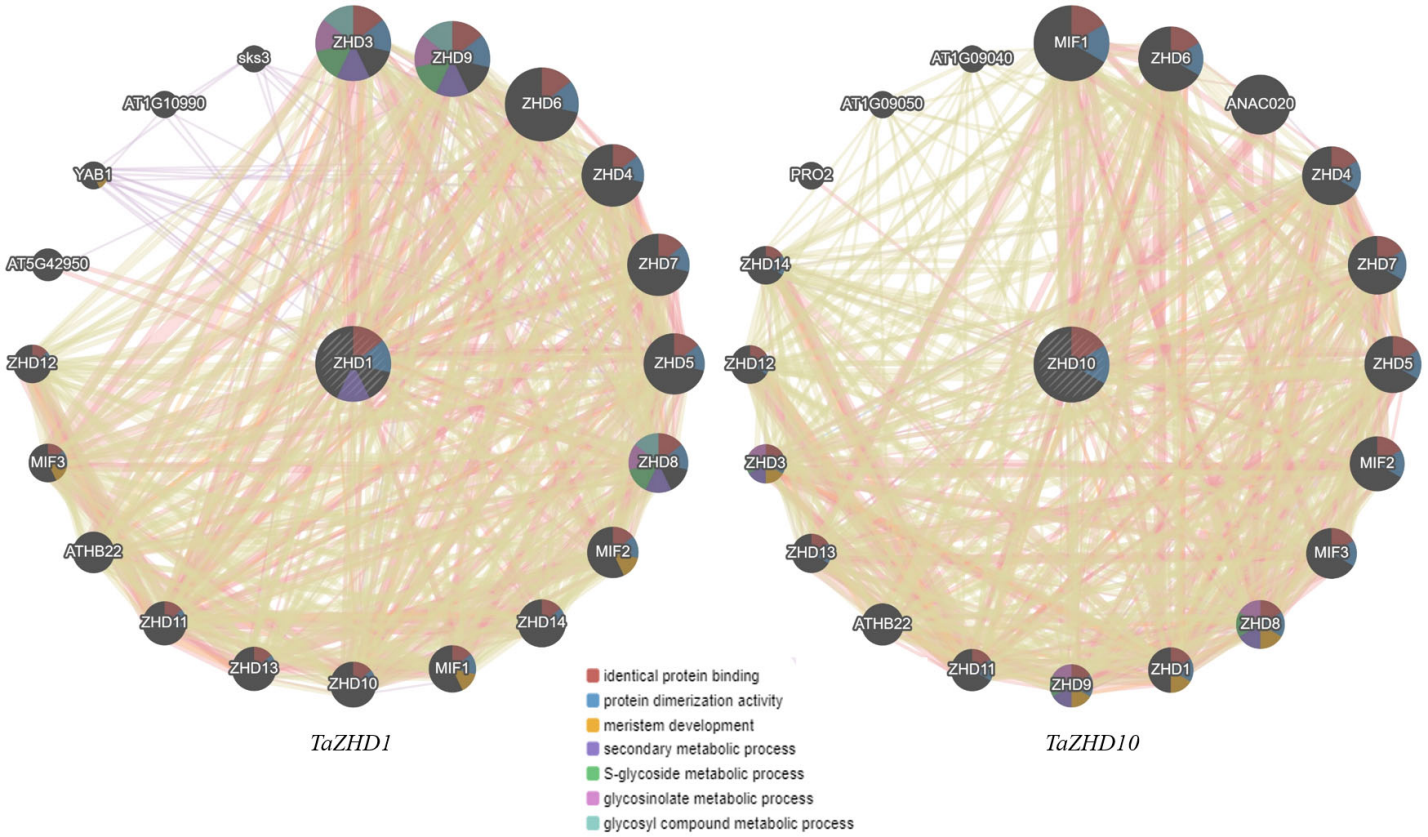
Gene interaction network of candidate leaf rolling *ZHD* genes with their regulatory partners taking *A. thaliana* as reference.

The predicted molecular interactome network revealed a set of zinc finger homeodomain (ZF-HD) IV family rich proteins having different regulatory partners based on the various parameters such as physical interactions, shared protein domains, genetic interactions, co-localization, and co-expression analysis (**Figure 9**). The interaction network clearly states that both *ZHD1* and *ZHD10* with their regulatory partners are involved in various metabolic and gene regulatory networks. Furthermore, up to 52.06% are engaged in close physical interactions, 21.72% are designated as candidate gene/transcription factors (TFs), 18.12% regulate the co-expression of particular genes, and 4.90% share their functional protein domains. Only 2.44% are involved in genetic interactions, while 0.76% is co-localized within similar cellular or sub-cellular compartments. The interaction network of both *ZHD1* and *ZHD10* share some common regulatory partners. The majority of them are dominated by the Zinc-finger homeodomain class proteins (ZHD3-9 and ZHD11-14), mini zinc finger proteins (MIF1-3), and *A. thaliana* homeobox protein 22 (ATHB22).

Apart from these, both *ZHD1* and *ZHD10* also exhibited significant interactions, suggesting that both might involve a common metabolic and gene regulatory network. Comprehensive analysis of the *ZHD1* interaction network further revealed that regulatory partners like GYF domain-containing protein (AT5G42950), axial regulatory YABBY1 (YAB1), SKU5 are similar 3 (sks3), an unknown protein (AT1G10990) does not exhibit significant interactions with *ZHD10* protein/genes. Similarly, the regulatory partners of *ZHD10* like NAC domain-containing protein 20 (ANAC020), Profilin-2 (PRO2), Arginine-glutamic acid dipeptide repeat protein (AT1G09050), and unknown protein (AT1G09040) are not showing any significant interactions with *ZHD1* protein/genes. Collectively, the resultant gene regulatory network integrates many genes controlling leaf rolling by coherent development of bulliform cells as presented in **Figure 9**.

### Epigenetic aspects of leaf rolling

We investigated the genomic DNA methylation levels in context of mCG, mCHG and mCHH, and found the wide coverage across the genic structure of both target genes. Consistent with the findings of qRT-PCR profiling, the DNA methylation profiles revealed heavily methylated and demethylated 5’ UTRs, introns, exons, and 3’UTRs regions for the mCG, mCHG and mCHH sequences which varied upon growth (stress) conditions (**Supplementary Table 7**). Under irrigated condition, there is no or little methylated and demethylated regions of target genes were observed. Unlike this, the mCG and mCHG sequence revealed significant methylated regions whereas the mCHH methylation of target genes was observed under drought condition. Congruent to this, variable demethylation levels in mCG, mCHG and mCHH sequences were recorded against the 24 hours of watering. We also observed significant differences in methylation patterns, specifically in the mCG context. *TaZHD10* revealed methylation while demethylation of *TaZHD1* at target exons was significantly more effective than *TaZHD10*. The results also confirmed findings of qRT-PCR profiling that the higher expression levels of *TaZHD10* than *TaZHD1* is a result of variable methylation-demethylation events. Taken together, methylome study suggested a critical role of DNA methylation in the regulation of *ZHD* genes, and also provides molecular insights into the epigenetic potential for controlling *TaZHD1* and *TaZHD10* mediated leaf rolling modulated by the drought-stress conditions.

## Discussion

Leaf architecture, such as shape and size, are critical morphological traits that affect the yield performance of the plant. Grass species such as maize and rice require the integration of critical developmental events to develop a single leaf from the founder cells, including differentiation, development, and elongation of leaf structures, leaf sheath, and leaf blade (Sakaguchi et al., 2010). Very young and tender leaf rolls inherently in the rice and maize when it is still enclosed with the sheath, is mainly *via* an unknown mechanism. Congruent to this, the mature leaves generally roll adaxially under stress conditions, probably through a mechanism controlled by the bulliform cells. Bulliform cells are thin-walled, large, highly vacuolated, and bulky cells present on the adaxial epidermis between the vascular bundles of the leaf blade (Fujino et al., 2008). It is proven that the adaxial bulliform cells lose turgor pressure under water stress, which results in leaf rolling. In contrast, when stress is relieved, bulliform cells maintain water potential and revive the surface tension. However, the actual physiology of bulliform cells in leaf rolling underwater deficiency is still to be proven (Li et al., 2016). Earlier studies advocate that bulliform cells might control the development of the young leaves, which are still rolled up in leaf sheath (Alvarez et al., 2008). It has also been proposed that bulliform cells could cause the erection of the leaf blade, reduce water loss, and increase stomatal resistance (Sakamoto et al., 2006). Apart from these, there are several factors which defines leaf rolling in plants. In general, leaf rolling caused by bulliform cells on adaxial leaf blade are regulated by the architecture (shape and size), number of bulliform cells and the different environmental factors (Alvarez et al., 2008).

Since the leaf architecture is a prominent agronomic trait in wheat breeding (Verma et al., 2020), the physio-bio-molecular mechanism of leaf rolling was of great interest, especially under the current environmental stress and nutrient limiting condition. Under drought stress, plant species like Arabidopsis, rice, wheat, maize, and sorghum exhibited characteristic leaf rolling as a tolerance response. In addition, leaf rolling has polygenic control with additive effects at target loci, especially in wheat (Sirault et al., 2008). There is scarce information about the genetic basis of leaf rolling in bread wheat (hexaploid), except for a report regarding stable QTL (*Qlr.nhv-5D.2*), associated with leaf rolling under moisture stress, located on the 5D chromosome (Verma et al., 2020). However, in emmer wheat (tetraploid) 11 QTLs related to leaf rolling were reported, significantly found to co-localized on 1A, 2A, 2B, 4B, 5A, 5B, 6A, 6B, 7A, and 7B chromosomes (Peleg et al., 2009).

Leaf rolling is a complex trait controlled by a myriad of genes. More than 70 QTLs/genes have been extensively studied in crops like rice and maize to regulate leaf rolling, but the complete molecular prospects of these complex traits are still not yet fully explored. The HD-Zip class IV family proteins are plant-specific transcription factors (TFs) classified based on sequence similarity into HD-Zip class I-IV family proteins (Gao et al., 2019). HD-Zip class IV family proteins (genes) occur in monocots (rice and maize), dicots (arabidopsis), and other plants (Bhattacharjee and Jain, 2013; Xu et al., 2014; Wang et al., 2016; Khatun et al., 2017; Abdullah et al., 2018; Yoon et al., 2020; Liu et al., 2021). These *ZHD* family genes are reported to involve in various morpho-physiological roles in plants, including vascular bundle development, light signaling, phytohormonal signaling, biogenesis of plant organs and stress responses (Tran et al., 2007, Liu et al., 2021). Arabidopsis contains 16 HD-Zip class IV family genes that play important roles during trichome development, root development, differentiation of epidermal cells, and anthocyanin accumulation (Xu F. et al., 2012; Gao et al., 2019). Similarly, rice contains 11 HD-Zip class IV family genes among which *OsRoc1* to *OsRoc8*, are specifically over-expressed in epidermal tissues of rice resulting in abaxial and/or adaxial leaf rolling *via* increased size and number of bulliform cells (Yun et al., 2010; Zou et al., 2011; Fang et al., 2021). More often, maize contains 17 HD-Zip class IV family genes among which *ZmOCL1, ZmOCL3, ZmOCL4*, and *ZmOCL5* are exclusively over-expressed in epidermal tissues whereas expression of *ZmOCL2* is restricted to the sub-epidermal tissues led to leaf rolling.

In addition, some *ZHD* genes are reported to have a significant modulatory role in defining organ identity under salt and dehydration stress (Wang et al., 2016; Abdullah et al., 2018). In arabidopsis, *AtZHD10* interacts with TZP proteins and regulates root development by hypocotyl elongation (Perrella et al., 2018). Relative expression of Arabidopsis ZHD protein (*AtZHD1*) is induced under salt and dehydration stress (Wang et al., 2016). It was later evidenced that ZHD TFs of *AtZHD1* can interact with promoters of *Early response to dehydration stress 1* (*ERD1*) at the zinc finger homeodomain recognition site (CACTAAATTGTCAC) and subsequently result in co-expression of both *ERD1* and *AtZHD1* under ABA responsiveness (Tran et al., 2007; Singh and Laxmi, 2015; Abdullah et al., 2018). Furthermore, it was also revealed that the NAC TF can also interact with ZHD-rich proteins that can simultaneously overexpress *NAC* and *ZHD* as a potential regulator of salt and drought tolerance in Arabidopsis (Hu et al., 2008). Finally, as reported in the present investigation, the results advocated that the identified *ZHD* genes encode a ZHD protein modulated by a HD-Zip class IV TFs (Ariel et al., 2007). As the potential regulator of drought tolerance (via leaf rolling) in various plant species, the HD-Zip class IV TFs can modulate leaf rolling *via* increased size and number of bulliform cells (Zou et al., 2011; Dong et al., 2019; Fang et al., 2021), particularly in the wheat crop.

In the present study, we reported for the first time two new wheat, *TaZHD1* and *TaZHD10* gene*s* containing a zinc finger homeodomain class homeobox transcription factors (TFs). We found that *TaZHD1* and *TaZHD10* are the closest orthologs of rice *OsZHD1* and *OsZHD10* and may play a key role in leaf rolling. Gene structure prediction revealed that the identified *TaZHD1* and *TaZHD10* possess the same exon-intron patterns, and the positions of the exons were highly conserved, which was consistent with earlier studies (Khatun et al., 2017; Abdullah et al., 2018). Besides, due to the consistent pattern of genes, the relative proportion of the length of exons in wheat was revealed to be highly coincident with earlier reports in rice, maize, tomato, cotton, and Arabidopsis (Xu G. et al., 2012; Khatun et al., 2017; Abdullah et al., 2018; Liu et al., 2021). The unique grouping of wheat and rice reflects the species-specific evolution of the *ZHD* family genes or could be either due to the domain or motifs-based phylogeny analysis. Undoubtedly, this is not surprising to consider the concept of remote-distance homology between the two co-evolving crop species (Moore and Purugganan, 2003). This result further demonstrates the reliability of evolutionary significance analysis for the independent classification of *ZHD* family genes. The *TaZHD1* and *TaZHD10* comprise ZF-HD dimer superfamily domains as functional domains presumably requisite for DNA-binding, suggesting that *TaZHD* genes act as potential transcription factors (Abu-Romman, 2014; Li et al., 2016). Moreover, potential domains or motifs and their gain or loss during gene duplication events might have contributed to the development of paralogous pairs of genes (Cannon et al., 2004; Xu G. et al., 2012).

The sequence analysis of identified chimeric ZHD proteins revealed wide variability in molecular weight, number of amino acids, GRAVY, aliphatic index, instability index, and isoelectric point (pI). The studied physicochemical properties might directly influence the stability of the chimeric protein (Chandra et al., 2021). Stability variations could be one of the driving factors for reduced leaf lamina joint angle with erect and rolled leaf behavior of the ZHD proteins while accumulation in sub-cellular compartments (i.e., nucleus) of the plants (Liu et al., 2021).

*Cis-*acting regulatory elements are a regulatory component of promoter regions of many genes that provide clues for stress responsiveness, light-responsiveness, hormonal responsiveness, growth and development, and other gene-specific expression patterns under specific environmental and growth conditions (Yun et al., 2010; Xu F. et al., 2012; Abdullah et al., 2018; Chandra et al., 2021). As reported in earlier studies, we predicted many *Cis*-regulatory elements in *TaZHD1* and *TaZHD10* promoter regions. We categorized these *Cis-*acting regulatory elements into three major groups based on their involvement in metabolic and gene regulation in the plant, including a) light-responsive, b) stress-responsive, c) hormonal responsive, d) involved in growth and developmental processes, and e) unknown function (Mohanty et al., 2012). CGTCA-motif and TGACG-motif are involved in (Methyl-Jasmonate) MeJA-responsive expression of genes (Abdullah et al., 2018). *Cis-*acting regulatory elements like Sp1 and G-box play a specific role as light-responsive elements, whereas GC-motif is an enhancer-like element involved in anoxic specific inducibility of target genes (Ezer et al., 2017; Liu et al., 2021). Besides zinc finger, the presence of numerous *Cis*-acting regulatory elements, including MYC (myelocytomatosis oncogene), CGTCA-motif, TGACG-motif, ABRE (ABA-responsive element), CCAAT-box, DRE core (dehydration-responsive element) and MYB (myeloblastosis oncogene) recognition site, suggesting the existence of multiple regulatory mechanisms in response to drought stress (Singh and Laxmi, 2015; Baldoni et al., 2015; Khatun et al., 2017; Dong et al., 2019; Chandra et al., 2021; Liu et al., 2021), that resulted in leaf rolling in plants (Dong et al., 2019). Additionally, the CCAAT-box contains MYBH1 binding sites that regulate various abiotic stresses and developmental processes (Laloum et al., 2013). Correspondingly, MYC controls plant growth and jasmonate (JA) induced defence gene activation (Yun et al., 2010; Singh and Laxmi, 2015). In contrast, the specific role of other *Cis-*acting regulatory elements like STRE (stress-responsive element), as-1, A-box, CCGTCC motif, and CCGTCC-box are yet to be defined (Xu F. et al., 2012; Gubert et al., 2014; Yoon et al., 2020; Liu et al., 2021). The drought responsive *Cis-*acting regulatory elements in the genes are consistent with their drought-responsive up-regulation during course of leaf rolling in plants.

Moreover, some reports elucidated that miRNAs can also play an important role while defining leaf differentiation by post-transcriptional silencing of target genes (Moon and Hake, 2011; Xu et al., 2018; Liebsch and Palatnik, 2020). For example, overexpression of *OsAGO7* resulted in adaxial leaf rolling in rice, which is knocked down by RNAi- the silencing mechanism. The transgenic plant exhibited significant pleiotropic defects resulting in phenotypic development like low plant height and narrow and erect rolled leaves (Shi et al., 2007; Wu et al., 2009). Additionally, miRNA160 and miRNA166 have played an important role in leaf rolling *via* xylem differentiation and reduced stomatal conductance by targeting *OsARF18* and *OsHB4*, respectively (Huang et al., 2016; Zhang et al., 2018). Thus, as reported in earlier studies, some other miRNAs like tae-miR1130b-3p, tae-miR531, tae-miR9666a-3p, tae-miR9664-3p, and tae-miR9672b can indirectly affect the leaf rolling by targeting *TaZHD1* and *TaZHD10*.

In the era of modern biological sciences, gene interaction network analysis through system biology has become an appealing research topic. Interactome networks render the functioning and offer a possible interaction of particular proteins within a cell to define any metabolic process (Yun et al., 2010; Borrill et al., 2016; Liu et al., 2021). Ever since, because of the unavailability of molecular interactome data of wheat in the public domain, we have used Arabidopsis interactome databases to predict the possible interaction of *TaZHD1* and *TaZHD10*. The results showed that *TaZHD1* might interact with the *TaZHD10* (**Figure 8**), which was congruent with our other results. The ZHD class IV transcription factors (TFs) can activate or suppress the expression of downstream target genes, thus regulating the important metabolic and gene regulatory networks (Tran et al., 2007; Abu-Romman, 2014; Abdullah et al., 2018). Therefore, we used available RNA sequence datasets to quantify the relative expression of *TaZHD1* and *TaZHD10* to explore the functional aspects. The result revealed the transcriptional expression of the identified gene, coinciding with earlier reports in rice confirming the paralogous pair of genes evolved through common gene duplication events (Moore and Purugganan, 2003).

qRT-PCR expression profiling of candidate genes in flag leaf tissue further revealed significant up-regulation or down-regulation consistent with other reported genes, *OsZHD1, OsZHD2, OsZHD10, YABBY1-7, SRL1*, and *ROC5* in rice and maize (Ohmori et al., 2011; Zou et al., 2011; Xiang et al., 2012; Xu et al., 2014). In present study, differential expression profiling under the drought-induced condition in the broadest section of young flag leaves was found significantly variable in the extreme RILs compared to parents (NI5439 and HD2012). The D-13 and D-65 RILs having higher leaf rolling than parents have highest expression of both the transcripts at both the stage and after adding water got down-regulated. Similarly, both transcripts in low leaf rolling (RIL D-7 and RIL D-9) were initially up-regulated under drought conditions (2-4 folds’ vs irrigated), and then down-regulated upon 24 HAW (1-3 folds vs drought). This result clearly states the role of *TaZHD1* and *TaZHD10* in leaf rolling in wheat. Unlike this, the study also reports co-expression of *TaZHD1* with *TaZHD10* under drought-induced conditions. It could be because of co-evolution of multiple or duplicate copy of gene as a phenomenon of stress tolerance mechanism. Our result was consistent with Hoffmann and Hercus (2000), reported environmental stressors as a key evolutionary forces for co-evolution of multiple or duplicate copy of gene. Not much earlier, significance of gene-duplication events and cross-talk between co-evolved genes was extensively studied in plants, specifically for salinity and drought tolerance in Arabidopsis (Panchy et al., 2016).

Overexpression of *TaZHD1* and *TaZHD10* can induce abaxial or adaxial curled leaf. *TaZHD1* and *TaZHD10* were the only homologs of *OsZHD1* and *OsZHD10* in rice, and its overexpression can also tempt abaxial or adaxial curled leaf. Similarly, the overexpression of *OsZHD1* with the closest homolog *OsZHD2*, results in induced leaf rolling by increasing the number and size of the bulliform cells at their abaxial surface (Xu et al., 2014; Zhu et al., 2017; Yoon et al., 2020). More often, a similar role was displayed by *ZmZHD10*, which exhibits characteristic bulliform cells shrinkage during drought, heat, and salt tolerance (Abdullah et al., 2018). These genes were stated to affect the differentiation of bulliform cells in leaf blades that result in leaf rolling in plants (Zou et al., 2011; Xu et al., 2014; Yang et al., 2016; Li et al., 2019). Together, the morphological pattern of the rolled leaf was perfectly matched with the expression patterns of studied genes, further suggesting that the *ZHD* class IV transcription factors (TFs) genes might act as an upstream regulator of bulliform cell differentiation necessary for leaf rolling. The study also indicated the pleiotropic role of annotated *ZHD* genes in wheat. Since, it was already reported in arabidopsis, rice, maize, tomato, and soybean that the *ZHD* family genes are involved in multiple plant developmental processes and stress tolerance (Xu et al., 2014; Wang et al., 2016; Liu et al., 2019; Yoon et al., 2020). So, likely the reported *TaZHD1* and *TaZHD10* could also be involved in other unknown functions in wheat *via* independent mechanisms or interaction in addition to leaf rolling.

Plant responses to different biotic and abiotic stress include various metabolic and genic reprogramming which are highly important for priming regulation of gene expression. More recently, evidence suggested the epigenetic regulation of gene expression at transcriptional and post-transcriptional level in response to abiotic stress (Kou et al., 2011; Yong-Villalobos et al., 2015). Different biotic and abiotic stresses can act as a stimulus and lead to alteration in genic expression by various epigenetic mechanisms like DNA methylation, histone modifications, nucleosome positioning, and miRNA (Saraswat et al., 2017; Guo et al., 2019). Among these epigenomics marks DNA methylation and histone modifications, are the most studied events in plants for exploration of epigenetic regulation of target genes (Hudson and Buck-Koehntop, 2018). For example, *A. thaliana ZHD* like i.e, *ASYMMETRIC LEAVES (AS2)* and Leaf Polarity gene *ETTIN/AUXIN RESPONSIVE FACTORS* (*ETT*/*ARF3*) are repressed due to CpG methylation resulted in altered leaf adaxial-abaxial development (Vial-Pradel et al., 2018). Similarly, histone modifications are a vital component of epigenetic mechanisms and are reported to have a pivotal role in controlling leaf differentiation (Xu et al., 2018). Since *TaZHD1* and *TaZHD10* are members of the zinc finger homeodomain (ZF-HD) IV family gene, they can play a pivotal role in gene expression *via* DNA methylation. Thus, differential expression patterns of *TaZHD1* and *TaZHD10* in flag leaf tissues under a drought-induced environment finally indicated its epigenetic aspects concerning the regulation of metabolic and gene regulatory networks involved in leaf rolling in wheat crops.

**Figure 10:**
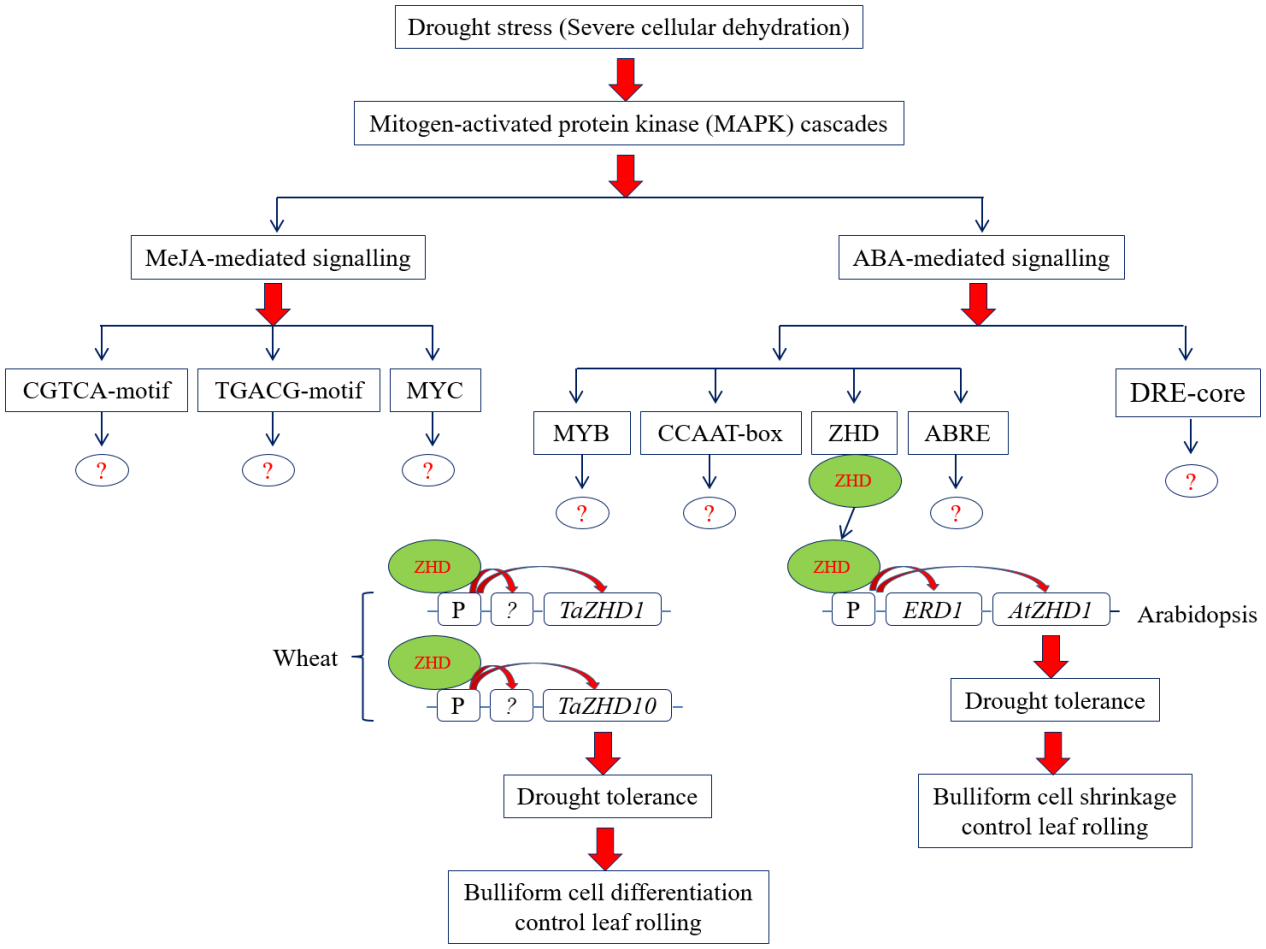
Proposed metabolic and gene regulatory model of leaf rolling under severe drought stress condition in wheat.

Conversely, the study further calls for systematic investigation would be necessary to extrapolate the functions of wheat *TaZHD1* and *TaZHD10* in leaf rolling by modern multi-omics technologies. In addition, we cannot exclude the prospect that the variation in the intergenic regions of the mapped QTL (*Qlr.nhv-5D.2*) interval may lead to a foundation for leaf rolling. A hypothetical metabolic and gene regulatory model that describes how identified *TaZHD1* and *TaZHD10* are involved in the leaf rolling under severe drought stress in wheat is presented in **Figure 10**. Besides all, the study further suggested that a complex metabolic and gene regulatory network might be involved in the leaf rolling in this important food crop. Finally, the present investigation can offer a valuable foundation for future functional research of other *TaZHD* genes or transcription factors for breeding wheat varieties tolerant to leaf rolling.

## Conclusion

This is the first comprehensive study that resulted in identification and characterization of two new leaf rolling genes, namely *TaZHD1* and *TaZHD10* in wheat, using a comparative genomics approach. This gene belongs to the zinc finger homeodomain (ZF-HD) class IV family and encodes a ZF-HD dimer superfamily domain-containing protein. The transcriptome-wide differential expression profiling resulted in many fold up-regulations of *TaZHD1* and *TaZHD10* in drought-induced leaf tissues. The result was further validated by qRT-PCR analysis which also demonstrated their significant upregulation upon drought while down-regulated upon 24 HAW. The contrasting modulation of these genes under a drought-induced environment and the available reports of its epigenetic behavior might provoke erect and rolled leaves. Overall, the substantiate validation of *TaZHD1* and *TaZHD10* suggested their functional redundancy and pinpoint importance in leaf rolling by regulating bulliform cells differentiation. However, the study also calls for a further comprehensive investigation to decipher the knowledge gaps concerning the ZHD proteins as a key regulator of bulliform cells activity and their possible molecular networks underlying leaf rolling in this important cereal crop.

## Data availability statement

The original contributions presented in the study are included in the article/**Supplementary Material**. Further inquiries can be directed to the corresponding authors.

## Author contributions

V and SJ conceptualized the research. SJ, V, and AC designed the experiments. V, MN, NM and SJ contributed experimental materials; AC and PA executed lab experiments and data collection; AC, SJ and V analyzed and interpreted data; AC and SJ wrote the manuscript. All authors contributed to the article and approved the submitted version.

## Funding

This work was funded by ICAR, Govt. of India sponsored project entitled “Incentivizing research in agriculture; Project IV: Molecular genetic analysis of resistance/tolerance to different stresses in rice, wheat, chickpea and mustard including sheath blight complex genomics - wheat component” (Project no.- 15613160025).

## Acknowledgments

The authors acknowledge ICAR sponsored project “Incentivizing research in agriculture” for funding and ICAR-Indian Agricultural Research Institute, New Delhi for providing all the necessary facilities.

## Conflict of interest

The authors declare that the research was conducted in the absence of any commercial or financial relationships that could be construed as a potential conflict of interest.

## Publisher’s note

All claims expressed in this article are solely those of the authors and do not necessarily represent those of their affiliated organizations, or those of the publisher, the editors and the reviewers. Any product that may be evaluated in this article, or claim that may be made by its manufacturer, is not guaranteed or endorsed by the publisher.

## Abbreviations

ZHD, Zinc finger-homeodomain; TFs, Transcriptional factors; RILs, Recombinant inbred lines; OCL, Outer cell layer; Roc, Rice outermost cell-specific; RAP-DB, Rice Annotation Project Database; QTL, Quantitative trait loci; NCBI, National Center for Biotechnology Information; BLAST, Basic Local Alignment Search Tool; ICAR, Indian Council of Agricultural Research; FYM, Farmyard manure; qRT-PCR, Quantitative real-time polymerase chain reaction.
