# Supplementary material for: Leaf rolling in bread wheat (Triticum aestivum L.) is controlled by the upregulation of a pair of closely linked/duplicate zinc finger homeodomain class transcription factors during moisture stress conditions
Source: Front Plant Sci. 2022 Nov 22;13:1038881. doi: 10.3389/fpls.2022.1038881 (PMC9723156; doi:10.3389/fpls.2022.1038881)
Supplement: Supplementary file 1 [file DataSheet_1.docx]

**Supplementary Table 1: Primer pairs used for quantitative real time PCR analysis**

| **S. No.** | **Primer ID** | **Sequence (5’------>3’)** | **Primer**  **Length (bp)** | **Tm** | **% GC** | **Product**  **Length (bp)** |
| --- | --- | --- | --- | --- | --- | --- |
|  | TaActin_F | GGACTTACAAAGGGGACTTG | 20 | 56 | 50.0 | 119 |
|  | TaActin_R | GAACCGAGACTGATTTTCCT | 20 |  | 45.0 |  |
|  | TaZHD1_F | GTTCTCGCCCTACTACCGC | 19 | 56 | 63.1 | 295 |
|  | TaZHD1_R | CTTCTGCTCCTGGGTGAACT | 20 |  | 55.0 |  |
|  | TaZHD10_F | CTGCTCTCTCTCAACTCCA | 19 | 56 | 52.63 | 288 |
|  | TaZHD10_R | GTAGTTGTGCTTGTTGTTGT | 20 |  | 40.00 |  |

**Supplementary Table 2: Details of predicted ORFs, physicochemical characteristics, and subcellular localization of identified leaf rolling proteins in wheat and its orthologs**

| **S. No.** | **Candidate Gene** | **Predicted best ORFs** | | | **Physicochemical characteristics** | | | | | **Subcellular localization** | | |
| --- | --- | --- | --- | --- | --- | --- | --- | --- | --- | --- | --- | --- |
|  |  | **Start** | **Stop** | **Length (bp/aa)** | **Molecular weight (kDa)** | **pI** | **Instability index** | **Aliphatic index** | **GRAVY** | **GO-id** | **GO-term** | **Score** |
|  | *OsZHD1* | 1385 | 432 | 954/387 | 40515.65 | 8.71 | Unstable (70.02) | 56.18 | -0.515 | GO:0005634 | Nucleus | 1 |
|  | *OsZHD10* | 286 | 1302 | 1017/338 | 35248.68 | 8.18 | Unstable (70.58) | 61.45 | -0.491 | GO:0005634 | Nucleus | 1 |
|  | *ZmZHD10* | 154 | 1254 | 1101/366 | 38269.08 | 8.63 | Unstable (70.78) | 62.65 | -0.382 | GO:0005634 | Nucleus | 1 |
|  | *TdZFH9a* | 214 | 1359 | 1146/381 | 39975.06 | 8.71 | Unstable (71.57) | 56.27 | -0.533 | GO:0005615; GO:0005634 | Extracellular space; nucleus | 0.64 |
|  | *TdZFH9b* | 131 | 1303 | 1173/390 | 40909.15 | 8.20 | Unstable (77.22) | 54.72 | -0.542 | GO:0005615; GO:0005634 | Extracellular space; nucleus | 0.67 |
|  | *TaZHD1* | 175 | 1338 | 1164/387 | 40511.66 | 8.71 | Unstable (71.85) | 56.18 | -0.517 | GO:0005634 | Nucleus | 1 |
|  | *TaZHD10* | 953 | >3 | 951/316 | 30966.64 | 7.01 | Unstable (55.62) | 50.61 | -0.534 | GO:0005634 | Nucleus | 1 |

**Supplementary Table 3: Details of discovered best motifs of identified leaf rolling protein in wheat and its homologs**

| **S. No.** | **Motif ID** | **Conserved sequences** | ***E*-value** | **Width** | **Log-Likelihood Ratio** | **Information Content** | **Relative Entropy** | **Bayes Threshold** | **Sites in chimeric proteins** |
| --- | --- | --- | --- | --- | --- | --- | --- | --- | --- |
|  | Motif-I | EQKQRMQELSERLGWRLQKRDEGVVDEWCRDIGVSKGVFKVWMHNNKHNY | 4.8e-231 | 50 | 1001 | 191.8 | 206.3 | 9.44501 | OsZHD1, OsZHD10, ZmZHD10, TdZFH9a, TdZFH9B, TaZHD1 and TaZHD10 |
|  | Motif-II | TYKECLKNHAAAIGAHAVDGCGEWMPVVELNTAD | 1.6e-116 | 34 | 610 | 127.8 | 125.8 | 8.29528 | OsZHD1, OsZHD10, ZmZHD10, TdZFH9a, TdZFH9B, TaZHD1 and TaZHD10 |
|  | Motif-III | SYKCAACGCHRNFHRLVMV | 1.7e-068 | 19 | 361 | 72.1 | 74.4 | 9.58747 | OsZHD1, OsZHD10, ZmZHD10, TdZFH9a, TdZFH9B, TaZHD1 and TaZHD10 |
|  | Motif-IV | MPMPMPATVLHGLPQRGHGQETPDDRLPGVDGDDSDSDSDGSEYDDERSV | 5.2e-040 | 50 | 415 | 212.4 | 199.5 | 9.89303 | TaZHD1, TdZFH9a and TdZFH9B |
|  | Motif-V | FNPSASHSSPAPTATGFNMNGTASSASTATTTATPTPIFAAGRKLNGASS | 3.4e-035 | 50 | 402 | 212.4 | 193.1 | 9.89303 | TaZHD1, TdZFH9a and TdZFH9B |
|  | Motif-VI | RKRFRTKFT | 1.3e-034 | 09 | 190 | 36.6 | 39.1 | 9.63057 | OsZHD1, OsZHD10, ZmZHD10, TdZFH9a, TdZFH9B, TaZHD1 and TaZHD10 |
|  | Motif-VII | QQPPPYISSAPHPHMLLSLNSSAPGAPQGQSRLPAQLSPATAPPPHGMM | 3.5e-028 | 49 | 382 | 209 | 183.9 | 8.40033 | TaZHD1, TdZFH9a and TdZFH9B |
|  | Motif-VIII | MEAMDVKYRPALYPNGSVKKLRQ | 3.0e-023 | 23 | 205 | 99.4 | 98.4 | 9.22831 | TaZHD1, TdZFH9a and TdZFH9B |
|  | Motif-IX | LGGHSARRSASAAASSAATTP | 3.0e-014 | 21 | 279 | 75.4 | 57.5 | 8.35378 | OsZHD1, OsZHD10, ZmZHD10, TdZFH9a, TdZFH9B, TaZHD1 and TaZHD10 |
|  | Motif-X | FSPTAMVPYGGVPHHQFSPYYRTPAGY | 6.4e-005 | 27 | 148 | 112.7 | 107.1 | 10.1376 | TaZHD10and OsZHD1 |
|  | Motif-XI | MDFDDHDDGDEEM | 1.0e-003 | 13 | 82 | 56.2 | 58.9 | 10.1991 | TaZHD10 and OsZHD1 |

**Supplementary Table 4: Details of superimposed template structure of identified leaf rolling gene (protein) in wheat**

| **S. No.** | **Superimposed Protein** | **Best Template structure** | **GMQE** | **QMEANDisCo Global** | **Identity** | **Method** | **Oligo State** | **Peptides** |
| --- | --- | --- | --- | --- | --- | --- | --- | --- |
|  | TaZHD10 | Homeobox domain of *Arabidopsis thaliana* hypothetical protein F22K18.140 | 0.10 | 0.62 ± 0.11 | 59.65 | SOLUTION NMR | Monomer | ZF-HD homeobox family protein: A |

**Supplementary Table 5: Description of Ramachandran plot structure assessment of identified leaf rolling gene (protein) in wheat**

| **S. No.** | **Superimposed protein** | **MolProbity score** | **Clash score** | **Ramachandran favoured** | **Ramachandran outliers** | **Rotamer outliers** | **C-Beta deviations** | **Bad bonds** | **Bad angles** |
| --- | --- | --- | --- | --- | --- | --- | --- | --- | --- |
|  | OsZHD1 | 2.70 | 3.85 | 83.33% | 3.33% | 12.73% | 1 | 0/526 | 7/699 |
|  | OsZHD10 | 2.84 | 7.63 | 86.44% | 3.39% | 10.91% | 2 | 1/535 | 12/712 |
|  | TdZFH9a | 2.84 | 7.63 | 86.44% | 3.39% | 10.91% | 2 | 1/535 | 12/712 |
|  | TdZFH9b | 2.84 | 7.63 | 86.44% | 3.39% | 10.91% | 2 | 1/535 | 12/712 |
|  | ZmZHD10 | 2.71 | 3.00 | 81.67% | 5.00% | 15.38% | 1 | 0/506 | 7/674 |
|  | TaZHD1 | 2.30 | 0.96 | 86.67% | 1.67% | 14.81% | 1 | 0/530 | 10/705 |
|  | TaZHD10 | 2.84 | 7.63 | 86.44% | 3.39% | 10.91% | 2 | 1/535 | 12/712 |

**Supplementary Table 6: miRNAs targeting identified leaf rolling gene in wheat**

| **S.No.** | **Target candidate gene** | **miRNA_Acc.** | **Expectation (E)** | **Alignment** | **Target Description** | **Inhibition** | **Multiplicity** |
| --- | --- | --- | --- | --- | --- | --- | --- |
|  | *TaZHD1* | tae-miR1130b-3p | 5.0 | 1585-CGUCCGUCCGUAAUAGAAGA-1604 | N/A | Cleavage | 1 |
|  |  | tae-miR531 | 5.0 | 176-GUCAAGAAGCUCCGGCAGGCG-196 | N/A | Cleavage | 1 |
|  |  | tae-miR9666a-3p | 5.0 | 671-CCGCCGUCGUACAUCUCUUCUG-692 | N/A | Cleavage | 1 |
|  | *TaZHD10* | tae-miR9664-3p | 4.5 | 243-GCACGCCGUCGACGGCUGCGG-263 | N/A | Cleavage | 1 |
|  |  | tae-miR9672b | 5.0 | 134-CUCCUGGUGGCGGUGGUGGUG-154 | N/A | Cleavage | 1 |

**Supplementary Table 7: Details of DNA methylome targeting identified leaf rolling gene in wheat and its best possible match with *O. sativa***

| **S. No.** | **Candidate gene** | **Target rice ZHD gene** | **Location of repeats** | **Types of methylation** | **Methylation sites** | **Methylation score** |
| --- | --- | --- | --- | --- | --- | --- |
|  | *TaZHD1* | *LOC_Os09g29130* (*OsZHD1*) | 5^’^ UTR: NA  E_1_: (CGG)n-440129  3^’^ UTR: (CTAG)n-440130, and (GA)n-440131 | mCG | 6 | 5^’^ UTR (0.167 and 0.5); E_1_ (-0.334, -0.286, -0.2, and -0.167) |
|  |  |  |  | mCHG | 5 | 5^’^ UTR (0.167 and 0.5); E_1_ (-0.2); 3^’^ UTR (-0.059 and 0.032) |
|  |  |  |  | mCHH | 6 | 5^’^ UTR (0.25, 0.167 and 1); E_1_ (-0.2); 3^’^ UTR (-0.334 and -0.059) |
|  | *TaZHD10* | *LOC_Os08g34010*  (*OsZHD10*) | E_1_: NA  I_1_: (A)n-409547 and (CGG)n-409548  E_2_: (CGA)n-909549, (CGG)n-409550, and (CGG)n-409551  3^’^ UTR: (GGAGAA)n-409552 | mCG | 3 | E_2_ (-0.889, 0.833, and 1) |
|  |  |  |  | mCHG | 6 | I_1_ (-0.5, -1, and 0.334); E_2_ (-1, 0.714, and 1) |
|  |  |  |  | mCHH | 5 | I_1_ (-0.5, and -1); E_2_ (-1, 0.714, and 1) |

**Note**: E, Exon; I, Intron; UTRs, Un-translated regions; mCG, CG-methylation; mCHG, CHG-methylation; mCHH, CHH-methylation

**Supplementary figure**


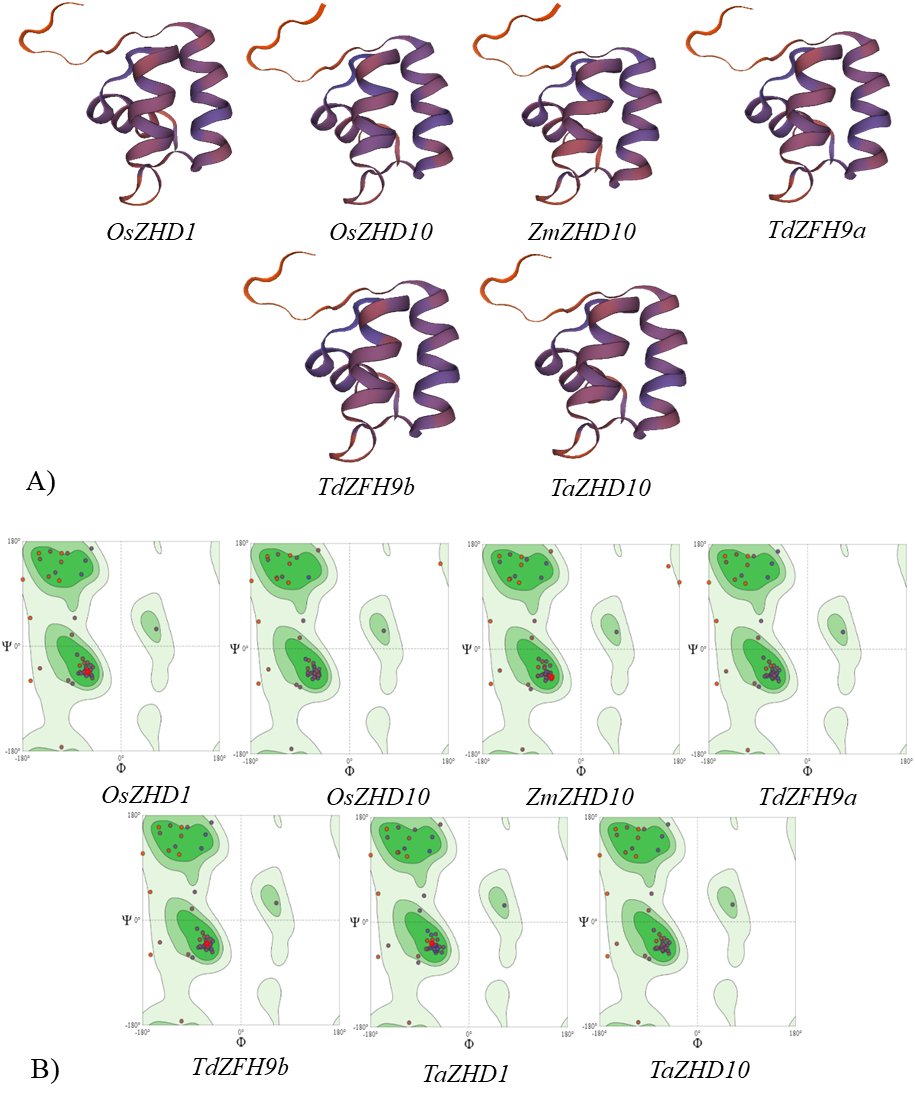


**Supplementary Figure 1** A) Homology modelling, and B) Ramachandran (phi/psi) plots of predicted leaf rolling genes (protein) of wheat and their orthologs
